# Identification of a dysregulated CircRNA-associated gene signature for predicting prognosis, immune landscape, and drug candidates in bladder cancer

**DOI:** 10.3389/fonc.2022.1018285

**Published:** 2022-10-10

**Authors:** Chong Shen, Zhi Li, Yinglang Zhang, Zhe Zhang, Zhouliang Wu, La Da, Shaobo Yang, Zejin Wang, Yu Zhang, Yunkai Qie, Gangjian Zhao, Yuda Lin, Shiwang Huang, Mingli Zhou, Hailong Hu

**Affiliations:** ^1^ Department of Urology, The Second Hospital of Tianjin Medical University, Tianjin, China; ^2^ Tianjin Key Laboratory of Urology, Tianjin Institute of Urology, The Second Hospital of Tianjin Medical University, Tianjin, China; ^3^ Department of Neuromuscular Diseases, Third Hospital of Hebei Medical University, Shijiazhuang, China

**Keywords:** bladder cancer, CircRNA-related prognostic signature, immune infiltration, immunotherapy, chemotherapy

## Abstract

Increasing evidences have demonstrated that circular RNA (circRNAs) plays a an essential regulatory role in initiation, progression and immunotherapy resistance of various cancers. However, circRNAs have rarely been studied in bladder cancer (BCa). The purpose of this research is to explore new circRNAs and their potential mechanisms in BCa. A novel ceRNA-regulated network, including 87 differentially expressed circRNAs (DE-circRNAs), 126 DE-miRNAs, and 217 DE-mRNAs was constructed to better understanding the biological processes using Cytoscape 3.7.1 based on our previously high-throughput circRNA sequencing and five GEO datasets. Subsequently, five randomly selected circRNAs (upregulated circ_0001681; downregulated circ_0000643, circ_0001798, circ_0006117 and circ_0067900) in 20 pairs of BCa and paracancerous tissues were confirmed using qRT-PCR. Functional analysis results determined that 772 GO functions and 32 KEGG pathways were enriched in the ceRNA network. Ten genes (PFKFB4, EDNRA, GSN, GAS1, PAPPA, DTL, TGFBI, PRSS8, RGS1 and TCF4) were selected for signature construction among the ceRNA network. The Human Protein Atlas (HPA) expression of these genes were consistent with the above sequencing data. Notably, the model was validated in multiple external datasets (GSE13507, GSE31684, GSE48075, IMvigor210 and GSE32894). The immune-infiltration was evaluated by 7 published algorithms (i.e., TIMER, CIBERSORT, CIBERSORT-ABS, QUANTISEQ, MCPCOUNTER, XCELL and EPIC). Next, Correlations between riskscore or risk groups and clinicopathological data, overall survival, recognized immunoregulatory cells or common chemotherapeutic agents of BCa patients were performed using wilcox rank test, chi-square test, cox regression and spearman’s correlation analysis; and, these results are significant. According to R package “GSVA” and “clusterProfiler”, the most significantly enriched HALLMARK and KEGG pathway was separately the ‘Epithelial Mesenchymal Transition’ and ‘Ecm Receptor Interaction’ in the high- vs. low-risk group. Additionally, the functional experiments *in vitro* also revealed that the overexpression of has_circ_0067900 significantly impaired the proliferation, migration, and invasion capacities of BCa cells. Collectively, the results of the current study provide a novel landscape of circRNA-associated ceRNA-regulated network in BCa. The ceRNA-associated gene model which was constructed presented a high predictive performance for the prognosis, immunotherapeutic responsiveness, and chemotherapeutic sensitivity of BCa. And, has_circ_0067900 was originally proposed as tumor suppressor for patients with BCa.

## Introduction

Bladder cancer (BCa) is the 10th most common malignancies in the world, with approximately 573,000 new cases and 213,000 fatal cases in 2020 (1). Unfortunately, despite advances in current clinical treatment for BCa, including surgical techniques, adjuvant/neoadjuvant chemoradiotherapies, radiotherapy, molecular targeted therapy and immune therapy, the recurrence and metastasis rates of BCa remain high, and the survival prognosis and the quality of life of some patients has not been significantly improved (2, 3). Therefore, the investigations of molecular mechanisms that contribute to above these phenomena, especially chemoresistance and immunotherapy resistance is eagerly explored.

Recently, circular RNA (circRNA) may be intimately associated with the recurrence, progression, chemoresistance, the regulation of immune microenvironment and the survival outcome for multiple cancers (4–9). For example, Chen et al. reveal that circ-ERBIN promotes the proliferation, invasion, angiogenesis and metastasis of colorectal cancer (CRC) through targeting miR-138-5p and miR-125a-5p, and thereby synergistically boosts the expression of the 4EBP-1, which next reinforces HIF-1α protein expression and activation of HIF-1α pathway (6). Recent researches showed that circ-VANGL1 was identified as a sponge of miR-605-3p targeting VANGL1 In BCa (10), Circ-ZEB1.33 *via* ponging miR-200a-3p and upregulating CDK6 in human hepatocellular carcinoma (11), circ_0020710 by targeting miR-370-3p/CXCL12 axis in melanoma (9), and circ0093740 through modulating miR-136/145-DNMT3A axis in Wilms Tumor (12), consequently contributes to cancer cells growth, migration, invasion or metastasis. It has been reported that circ-MTHFD1L efficiently sponges to miR-615-3p, debilitates the inhibitory effect of miR-615-3p on RPN6, contributes to gemcitabine chemoresistance in pancreatic cancer (PDAC) cells, and serve as a novel biomarker for diagnosing PDAC chemoresistance (13). In 2022, Hu et al. found that the circFARP1 overexpression in CAFs promoted gemcitabine resistance in PDAC by increasing the expression and secretion of leukemia inhibitory factor in CAFs and thus activating the STAT3 signaling pathway in PDAC cells (14). And notably, our previous research has demonstrated that up-regulation of circASXL1 in BCa relates with higher TNM classification and may independently predict poor overall survival (OS) for patients with BCa (15). Furthermore, our additional preliminary works illustrated that circ_0004826, circ_0077837 and circ_0030586 might act as tumor suppressors in BCa and act as a potential biomarker for the prognosis, diagnosis and therapy of BCa (16, 17).

It has been showed that circRNA CDR1as acted as an oncogene, promoted NSCLC progression and maintained the undifferentiated status of mesenchymal stem cells by targeting miR-219a-5p/SOX5 axis (18). In the same year, Zhao et al. revealed that knockdown of circCDR1as sensitized Cisplatin-resistant NSCLC cells to cisplatin and weakened cell stemness characteristics by targeting miR-641/HOXA9 axis (19). In the same way, Yang et al. also reported that inhibition of circCDR1as sensitized breast cancer cells to 5-fluorouracil (5-FU) through up-regulating miR-7 (20).

The close connection between circRNAs and miRNAs or some proteins can help circRNA regulate immune responses in tumor through circRNA–miRNA–mRNA axis or modulate the stability of some proteins like p53 (5). In breast cancer, Rao et al. (21) indicated that circRNA–miRNA interactions participate in cancer progression and immune evasion by regulating the expression of CCND1, SKIL and CD46 genes. Recently, it has been reported that circRNAs strongly arouse immune signaling of RIG-I feedback-mediated (22). For example, Li et al. revealed that circNDUFB2 suppresses NSCLC progression through destabilizing IGF2BPs, and it is also recognized by RIG-I to activate RIG-I-MAVS signaling cascades and enroll immune cells into the tumor microenvironment for triggering anti-tumor immunity (23). In addition, dysregulation of circRNAs may serve as tumor antigen owing to their evolutionarily conserved, stable and specific properties (5), and play significant roles in the initiation, progression and metastasis of numerous cancers by tuning abundance in immune cells (i.e., Treg cells, CD8+ T-cell, plasma cells, M2 macrophages, cancer-related fibroblast, etc), pathological angiogenesis and antitumor Immunity (14, 24–27). Although there have been some previous achievements on circRNAs in BCa; however, the expression profiles and accurate molecular regulatory mechanisms of BCa-specific circRNAs, especially in the regulation of the tumor microenvironment, has not been well uncovered.

Thus, *via* high-throughput RNA sequencing (RNA-seq), we here explored the expression profiles of circRNAs and determined 87 significantly differentially expressed (DE) circRNAs, in five BCa samples compared to paired noncancerous peritumoral samples. Besides this, to investigate the underlying molecular mechanism of circRNAs in patients with BCa, we collected and analyzed additional five datasets (including three miRNA and two mRNA microarray data) from GEO databases, and then constructed a DEcircRNAs-DEmiRNAs-DEmRNAs competitive endogenous RNA (ceRNA) network following the strict ceRNA hypothesis. The 10 genes in the ceRNA network were selected and constructed a risk signature in the TCGA cohort using lasso-penalized cox regression analysis; later, the model was validated in multiple external datasets (GSE13507, GSE31684, GSE48075, IMvigor210 and GSE32894). Surprisingly, the ceRNA-associated gene model could accurately predict the clinicopathological features and the survival prognosis of patients with BCa. Moreover, the results showed that the ceRNA-associated signature was involved with the regulation of immune infiltration and immune checkpoints. Collectively, this study systematically analyzed the clinicopathologic features correlation, prognostic value, effects on the immune microenvironment and the underlying molecular mechanism of ceRNA-associated gene signature in BCa. Additionally, the results also indicated that has_circ_0067900 may act as a tumor suppressor, and promising biomarker for diagnostic and prognostic predication in BCa patients.

## Material and methods

### Clinical specimens

A total of 20 BCa and their matched adjacent non−tumorous tissues were collected between January 2021 and December 2021 at the Second Affiliated Hospital of Tianjin Medical University (China). The final diagnosis of each patient was confirmed histopathologically. In the current study, written informed consent was gained from every patient or their guardians based on the guidelines approved by the Medical Ethics Committee of the Second Hospital of Tianjin Medical University.

### Data collection and processing

The datasets used to constructed or verified the ceRNA-associated gene signature of BCa were from five different platforms: The Cancer Genome Atlas (TCGA) and Gene Expression Omnibus (GEO). According to TCGA database (https://portal.gdc.cancer.gov/repository), 406 BCa samples were downloaded, and included mRNA expression profile data and clinical information. Gene annotation was implemented through the Ensemble database. The microarray expression data of four GEO datasets, GSE13507 (n = 165), GSE31684 (n = 93), GSE48075 (n = 73), and GSE32894 (n = 224), were all quantile-normalized, and the genes were annotated in their respective microarray platform files GPL6102, GPL570, GPL6947, and GPL6947. The IMvigor210 mRNA-sequencing dataset, a cohort of 348 MIBC patients treated with Atezolizumab (PD-L1 inhibitor), was also used for validation of ceRNA-associated gene signature.

### CircRNA profiling analysis

Five pairs of BCa and adjacent noncancerous samples were sequenced by using an Illumina HiSeq 4000 sequencer. In short, paired-end reads were obtained from Illumina HiSeqTM 4000 sequencer (Illumina). After 3′ adaptor-trimming and low-quality reads were removed through cutadapt software (v1.9.3), the cleaned high-quality reads were aligned to the reference genome using STAR aligner (v2.5.1b) (28). Then, circRNAs were identified and annotated using the DCC software and the circBase database, respectively. The edgeR software (v3.16.5) was used to normalized and conducted for differentially expressed circRNA analysis (29). To be clear, the original sequencing data has been uploaded to NCBI SRA database, which can be accessed through series of SRA numbers SRP229539.

### Cell culture and lentiviral transfection

Human two bladder cancer cell lines (T24 and 253J−BV) were obtained from the Chinese Academy of Sciences Cell Bank (Shanghai, China). The T24 and 253J−BV cells were maintained in the RPMI−1640 medium (Biological Industries) supplemented with 10% fetal bovine serum (FBS; Gibco) and 1% penicillin-streptomycin (Gibco) in a humidified incubator containing 5% CO2 at 37˚C. The lentiviral vectors with has_circ_0067900/empty vector (NC) were synthesized by Hanbio Biotechnology Co., Ltd. After 24 hours of culture, cells were transfected with the lentiviral vectors. Expression of has_circ_0067900 was observed and determined 72 hours after transfection using an inverted microscope with fluorescence observation and quantitative real-time PCR.

### Prediction of miRNA–mRNA and circRNA–miRNA Interactions, and network construction

The potential circRNA–miRNA interactions were predicted using the Cancer-Specific CircRNA Database (CSCD) (http://gb.whu.edu.cn/CSCD/) and the Circular RNA Interactome (CircInteractome) (https://circinteractome.nia.nih.gov/), an online tool. We determined the regulatory potential miRNAs by taking the intersection of DE-circRNAs-targeting miRNAs and differential miRNA from three datasets (i.e., GSE11224, GSE113786 and GSE113740). Next, to infer the miRNA–mRNA interactions, various bioinformatics tools, such as miRTarBase (http://mirtarbase.cuhk.edu.cn/), miRDB (http://www.mirdb.org/miRDB/) and TargetScan (http://genes.mit.edu/targetscan/), were adopted. We identified the regulatory potential mRNAs by taking the intersection of aforementioned DE-miRNAs-targeting genes and differential mRNA from two datasets (i.e., GSE13507 and GSE37815). Briefly, to identify dysregulated ceRNA interactions, the regulatory relationships as well as expression associations among miRNA, circRNA, and mRNA were considered. Subsequently, Cytoscape 3.7.1 software was used to construct the circRNA-miRNA-mRNA interaction network.

### GO term enrichment and KEGG pathway analysis

To understand the underlying biological mechanisms of the ceRNA network, GO and KEGG enrichment analysis was conducted by the clusterProfiler R package. GO terms were divided into three domains: Biological Process (BP), Molecular Function (MF) and Cellular Component (CC). The GO plot package of the R software was applied to present the results of the GO and KEGG analyses. The GO and KEGG significantly enriched terms were screened with P <= 0.05 as threshold.

### Construction of the ceRNA-associated gene signature model and validation

Lasso-penalized cox regression analysis was conducted to establish the ceRNA-associated gene signature. The optimal values of penalty parameter lambda were determined *via* 10-fold cross-validation in the TCGA_BLCA training dataset. The risk score of BCa patients was calculated using the ceRNA-associated gene signature, (CoefficientmRNA1 * expression of mRNA1) + (CoefficientmRNA2 * expression of mRNA2) + ⋯ + (CoefficientmRNAn * expression of mRNAn). According to the median risk score as a cutoff value, 406 BCa patients in each cohort in the training cohort were divided into high- and low-risk groups. Kaplan-Meier (KM) survival and time-dependent receiver operational feature (ROC) and calibration curves were plotted by “survival”, “timeROC” and “RMS” R packages to evaluate the discrimination and calibration of the ceRNA-associated 10-gene model. Besides that, the ceRNA-associated 10-gene model was validated in five independent test cohort (i.e., GSE13507, GSE31684, GSE48075, IMvigor210 and GSE32894). Clinicopathological and survival data of these datasets was downloaded from the TCGA and GEO data portal and manually collected (**Supplementary File S1**). Furthermore, the 10 modeled genes expression in the signature was further validated by using The Human Protein Atlas database (HPA; **Figure 4**).

### The correlations between model and clinical traits; independent predictor; predictive nomogram construction

Wilcox test was carried out in investigating the clinical associations of the ceRNA-associated 10-genes riskscores group and various clinical factors. The Spearman rank correlation analysis was adopted to analyze the correlation between ceRNA-associated model riskscores and clinical characteristics of BCa patients. Univariate and multivariate cox regression analysis for OS were conducted to estimate whether the ceRNA-associated 10-genes model could be independent of other clinicopathological features (including gender, age, histologic grade, stage, depth of invasion, lymphatic metastasis, distant metastasis and risk score) (**Figure 6D**) for BCa patients. Besides, the nomogram and corresponding calibration plots was generated using the “RMS” R package.

### Correlations between the ceRNA-associated gene signature and multiple immune cells inferred by seven immune-infiltration algorithm

To explore the immune-infiltration in BCa, we employed the 7 immune-infiltration algorithm (TIMER, CIBERSORT, CIBERSORT-ABS, QUANTISEQ, MCPCOUNTER, XCELL and EPIC) to calculate the proportion of various immune cells and revealed the function of immune-infiltration *via* multiple strategies. The Wilcox rank-sum test were adopted to analyze the differences between the two riskscore groups; then, the results were visualized by heat maps through the R package “Pheatmap”. Correlations between riskscore and immune-infiltration cells were analyzed by the Spearman rank correlation analysis. Moreover, the tumor immune dysfunction and exclusion (TIDE) algorithm analysis, based on simulating tumor immune evasion mechanism, and partial immune checkpoint gene expression was applied to predict the immunotherapy response of each patient using Wilcox test based on TCGA_BLCA, IMvigor210 and GSE32894 datasets.

### Gene set enrichment analysis and chemosensitivity

To further understand and identify enriched cellular pathways associated with the 10-genes model in BCa, we applied the GSVA methods to assess the Hallmark gene sets activity from MSigDB (http://software.broadinstitute.org/gsea/msigdb) using R package “GSVA” based on TCGA_BLCA RNA-seq data. Similarly, R package clusterProfiler was used for GSEA to compare the different Hallmark or KEGG pathways between the two risk groups from TCGA_BLCA RNA-seq data. The R package “pRRophetic” was utilized to predict the sensitivity of two riskscore groups to Cisplatin, Docetaxel, Paclitaxel and Vinblastine.

### RNA extraction and quantitative reverse-transcription PCR

Extraction of total RNA from cell and tissue samples was collected with E.Z.N.A.™ Hp total RNA Kit (OMEGA). The cDNA was reversed from total RNA with RevertAid First Strand cDNA Synthesis Kit (Thermo Fisher Scientific,Rockford, IL, USA). Real-time quantitative PCR was utilized to assess the expression of circRNA, which was used by TOROGreen qPCR Master Mix (Toroivd). We validated the head-to-tail splicing of the circ_0067900 by Sanger sequencing. Furthermore, total RNA was treated with RNase-R+ (Epicentre) previously to cDNA synthesis to detect resistance of circ_0067900 to RNase-R digestion. The primer sequences applied in the present study are displayed in **Supplementary Table 1**. GAPDH was used as a control gene. The final results were analyzed using the 2^-ΔΔ^CT method.

### CCK-8 assay and clone formation assays

Following transfection, 1.5 × 10^3^ BC cells were seeded into the 96-well plate and next cultured at 37°C in 5% CO_2_ for 96 h. Each well was added with 90 μl new medium and 10 μl CCK-8 (Boster Bio). After 4 hours in dark, a VersaMax Microplate Reader was used to measure the optical density at 405 nm. For clone formation assay, 0.5 × 10^3^ BCa cells were plated into six-well plates and cultured for 10 days. Colonies were then fixed with 4% paraformaldehyde for 15 minutes, stained with 0.1% crystal violet (Solarbio) for 15 minutes and counted by ImageJ software version 1.8.0.

### Wound healing assay

Following transfection, the BC cells were cultured in 6 cm dishes till reaching 90%-95% confluency. The wound was scratched by using 200 μl tips and washed by PBS (Gibco). The photographs were taken at 0 h and 24 h to evaluate the proliferation and metastasis of BC cells.

### Transwell migration and invasion assays

For cell migration and invasion assays, 4 × 10^4^ cells were seeded with 200 μl of serum-free medium in the upper chamber (0.8 µm; Corning) and 700 µl complete medium in the lower chamber with (for invasion assays) or without (for migration assays) the Matrigel (Corning). Following incubation for 36 h, 4% paraformaldehyde (Sigma) and 0.1% crystal violet (Solarbio) were used to fix and stain the BC cells. Quantification of cell migration and invasion were done using olympus microscope (Olympus).

### Statistical analysis

Statistical analysis results are shown as mean ± standard deviation (SD). Comparisons between the two riskscore groups were evaluated with a chi-squared test for nominal variables and a nonparametric Wilcox test for continuous variables. Correlations were analyzed using Spearman’s rank correlation analysis. All reported p values were two-sided, and a p value lower than 0.05 was considered statistically significant. Data entry and calculation were done using the R software version 3.6.1 (R Core Team, 2019), IBM SPSS 24.0 (IBM Corp., 2016) and Microsoft Excel (Microsoft Corp., 2019).

## Results

### Identification of DE-circRNAs, DE-miRNAs, DE-mRNAs

**Figure 1:**
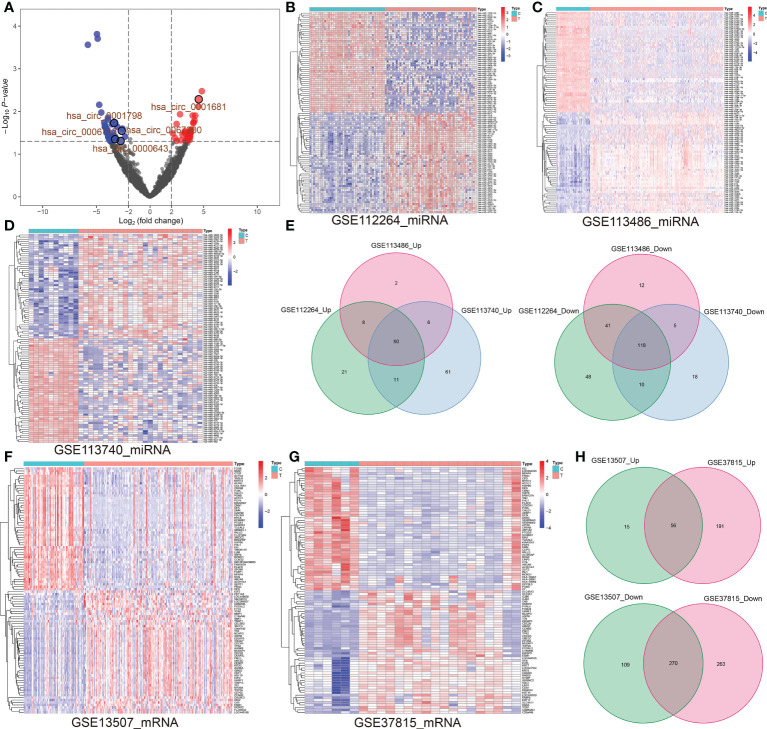
**(A)** Volcano map representing the up-regulated and down-regulated circRNAs in BCa vs. para-carcinoma tissues (log2(FC) ≥2 or ≤ -2; P< 0.05). **(B–D)** Based on GSE11224, GSE113786 and GSE113740 datasets, top 50 up-regulated and top 50 down-regulated miRNAs in BCa tissues vs. controls were shown. **(E)** Venn diagrams revealed the number of overlapping up-regulated (left) or down-regulated (right) miRNAs among GSE11224, GSE113786 and GSE113740. **(F, G)** Based on two datasets (GSE13507 and GSE37815), top 50 up-regulated and top 50 down-regulated miRNAs in BCa tissues vs. controls were exhibited. **(H)** Venn diagrams represent overlapping up or down-regulated genes between GSE13507 and GSE37815 datasets. circRNA, circular RNA; miRNA, microRNA; BCa, bladder cancer; FC, fold change.

Five pairs of BCa tissues and corresponding adjacent tissues were used to identify circRNA expression profiles by RNA-seq. Among these circRNAs, we observed 40 significantly upregulated circRNAs and 47 significantly downregulated circRNAs based on fold change more than 2 (log2(FC) > 2 or < -2) and P value lower than 0.05 (P < 0.05). Volcano plots comparing the expression of circRNAs in BCa tissues to adjacent normal tissues (**Figure 1A** **;** **Supplementary File S2**). Likewise, the differential miRNA expression analysis of GSE11224, GSE113786 and GSE113740 dataset respectively identified 317 (including 217 upregulated and 100 downregulated), 252 (including 76 upregulated and 176 downregulated) and 541 (including 289 upregulated and 252 downregulated) DE-miRNAs by limma R package (adjusted P < 0.05 and |log2(FC)| > 2) (**Supplementary File S3**). Heat maps were generated for these top 50 upregulated and downregulated DE-miRNAs (**Figures 1B–D**). Wayne diagram showing the 60 common upregulated and 118 common downregulated miRNAs in the three GEO databases (**Figure 1E**). Similarly, the differential expression analysis of GSE13507 and GSE37815 datasets respectively determined 450 and 780 DE-mRNAs using R package limma, the former included 71 upregulated and 379 downregulated DE-mRNAs, the latter had 533 and 247 (adjusted P < 0.05 and | log2(FC)| > 1) (**Supplementary File S4**). Heat maps of the top 50 up- and downregulated DE-mRNAs is presented in **Figures 1F, G**. Wayne analysis representing the 56 common upregulated and 270 common downregulated genes in the two datasets (**Figure 1H**).

### Prediction for the circRNA-miRNA-mRNA interactions and network visualization

**Figure 2:**
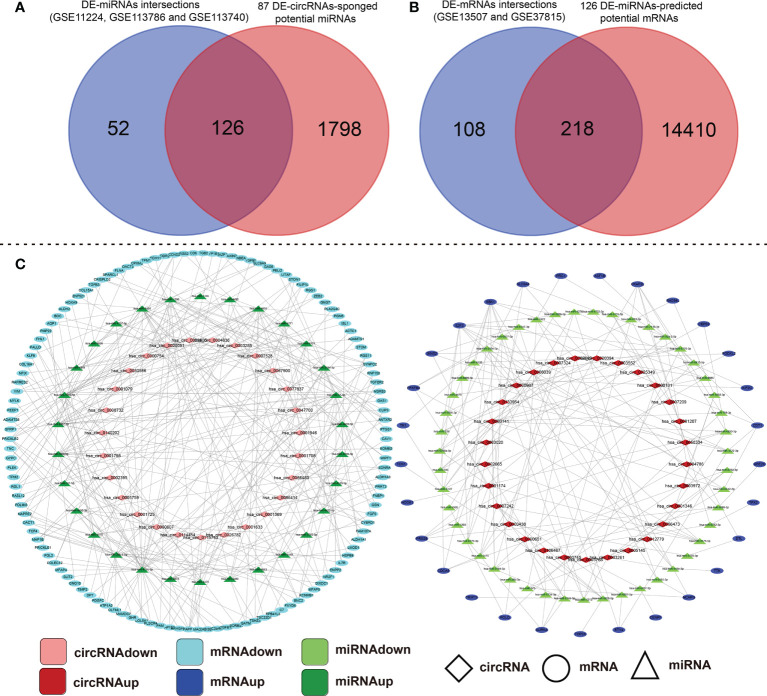
Construction analysis of the ceRNA network of the DE-circRNA, DE-miRNA, and DE-mRNA identified in BCa tissues compared with their adjacent non-cancerous tissues. **(A)** Intersection of DE-circRNA-targeting miRNAs and common DE-miRNAs of GSE11224, GSE113786 and GSE113740 datasets. **(B)** Conjoint analysis of the A-mentioned 126-intersection-DE-miRNA binding mRNA targets and DE-mRNA of GSE13507 and GSE37815 datasets. **(C)** The circRNA-miRNA-mRNA ceRNA network was established and visualized by Cytoscape 3.7.1 that based on the above data. ceRNA, competing endogenous RNA; circRNA, circular RNA; miRNA, microRNA; differentially expressed-, DE-.

A large number of studies have confirmed that circRNAs sponge miRNAs to influence the biological function of miRNAs on target genes. To further investigate the regulatory mechanism of circRNAs, we predicted the miRNA binding sites for the 87 DE-circRNAs using the CSCD and CircInteractome online database (**Supplementary File S5**). Next, 126 miRNAs were obtained by taking the intersection of the predicted potential miRNAs and common DE-miRNAs of the above three miRNAs datasets (**Figure 2A**). To predict downstream mRNAs on the 126 integrated DE-miRNAs, we screened out the intersection of mRNAs predicted by at least two popular prediction softwares such as miRTarBase, miRDB and TargetScan. Then, taking the intersection of predicted genes and common DE-mRNAs of the above two mRNAs datasets, 218 mRNAs were included and the other 14410 mRNAs were excluded (**Figure 2B**). Subsequently, based on aforementioned these data, we constructed two types of circRNA-miRNA-mRNA ceRNA regulatory networks and visualized them through cytoscape 3.7.1 software (**Figure 2C**). The above findings perfectly accorded with the “ceRNA network hypothesis”. Namely, there is an opposite differentially expressed trend in circRNA-miRNA or miRNA-mRNA interactions; however, the same tendency was seen in circRNA-mRNA.

To better understand the ceRNA network, we extracted and analyzed BCa expression profiles of miRNA, and mRNA from TCGA database. The 12 miRNAs in the intersection of ceRNA-associated DE-miRNAs and TCGA_BLCA miRNAs, include miR-17-3p, miR-191-5p, miR-769-3p, miR-29b-1-5p, miR-26b-3p, miR-4652-5p, miR-326, miR-6734-5p, miR-4732-3p, miR-520f-5p, miR-4444 and miR-3173-5p. Hence, correlations between these 12 miRNAs and multiple clinicopathological characteristics of BCa was assessed based on the TCGA database. Using median expression value of miRNAs as the cut-off point for the Chi-squared test or Fisher’s exact test, it was revealed that the low expression of miR-191-5p, miR-26b-3p and miR-17-3p was obviously correlated with tumor invasion depth, higher stage, higher histologic grade, older age, higher male ratio, especially the first two. Whereas, the high expression of miR-3173-5p was only related to a higher histologic grade (P < 0.01); and, the low expression of miR-4652-5p was only associated with a lymphatic metastasis (P < 0.01) (**Supplementary Table 2**).

The 115 common mRNAs were obtained by taking the intersection of ceRNA-associated DE-mRNAs and TCGA_BLCA mRNAs. In the later, we performed spearman correlation analysis between these 12 DE-miRNAs and 115 DE-mRNAs. The Sankey and network diagrams was drawn to visualize miRNA-mRNA co-expression relationships (**Supplementary Figures 1A, B**).

### Functional enrichment and the Lasso−penalized Cox regression analyses of these DE-mRNAs in the ceRNA network

**Figure 3:**
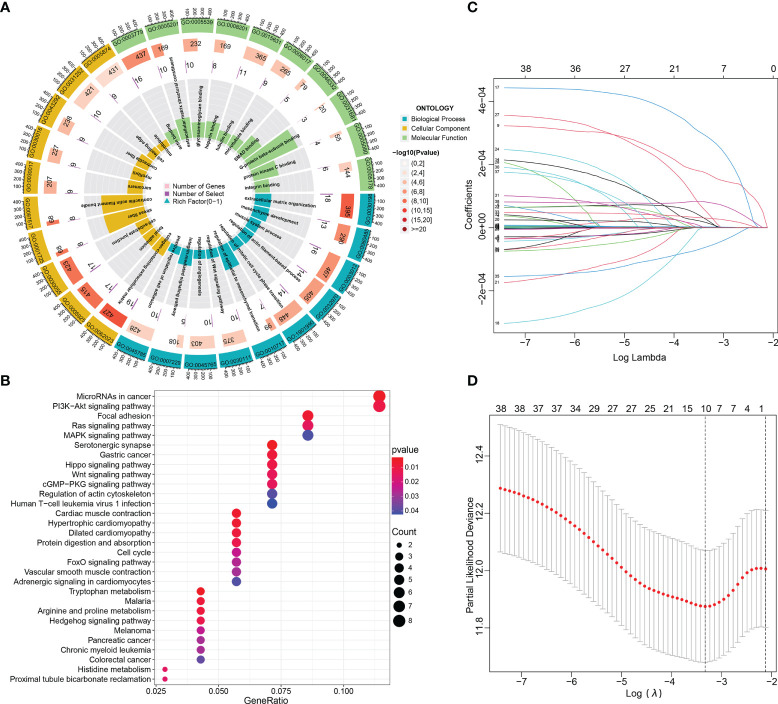
Functional enrichment and Lasso-penalized Cox regression analyses of these DE-mRNAs in the ceRNA network. **(A, B)** The GO function (BP, CC and MF) and KEGG pathway annotation analyses of DE-mRNAs in the ceRNA network. **(C, D)** Lasso regression analysis selected prognosis–related DE-mRNAs in the ceRNA network. ceRNA, competing endogenous RNA; BP, biological process; MF, molecular function; CC, cellular components; DE-, differentially expressed-.

To better clarify the functional pathways for the 87 DE-circRNAs in BCa, GO function annotations and KEGG pathway enrichment analyses of these 152 target DE-mRNAs in the ceRNA regulatory network above were performed. The target genes were analyzed with functional analysis, GO annotations and the KEGG pathway. In the GO analysis, we obtained 204 enriched results (143 BP, 45 CC and 16 MF) with adjusted p-value <0.05, which were shown in **Supplementary File S6**. The 10 significant and meaningful GO terms of BP (e.g., ‘regulation of epithelial to mesenchymal transition (GO:0010717)’, ‘regulation of mitotic cell cycle phase transition (GO:1901990)’ and ‘regulation of Wnt signaling pathway (GO:0030111)’, etc), MF (e.g., ‘actin binding (GO:0003779)’, ‘extracellular matrix structural constituent (GO:0005201)’ and ‘microtubule binding (GO:0008017)’, etc), and CC (e.g., ‘collagen-containing extracellular matrix (GO:0062023)’, and ‘focal adhesion (GO:0005925)’, etc) were exhibited in **Figure 3A**.

In KEGG pathway analysis, we got 32 results with p-value < 0.05, which were shown in **Supplementary File S7**. The dotplot in **Figure 3B** showed the results of the top 30 enrichment KEGG terms with P-values ranging from low to high; and of them, the five most enriched terms were “MicroRNAs in cancer”, “ PI3K−Akt signaling pathway”, “Focal adhesion”, “Ras signaling pathway” and “MAPK signaling pathway”. Subsequently, of these DE-mRNAs in the ceRNA network, Least absolute shrinkage and selection operator (Lasso) regression was applied to screen out the optimal gene combination for constructing the risk signature (**Figures 3C, D**).

### Construction and validation of the ceRNA-associated gene signature

**Figure 4:**
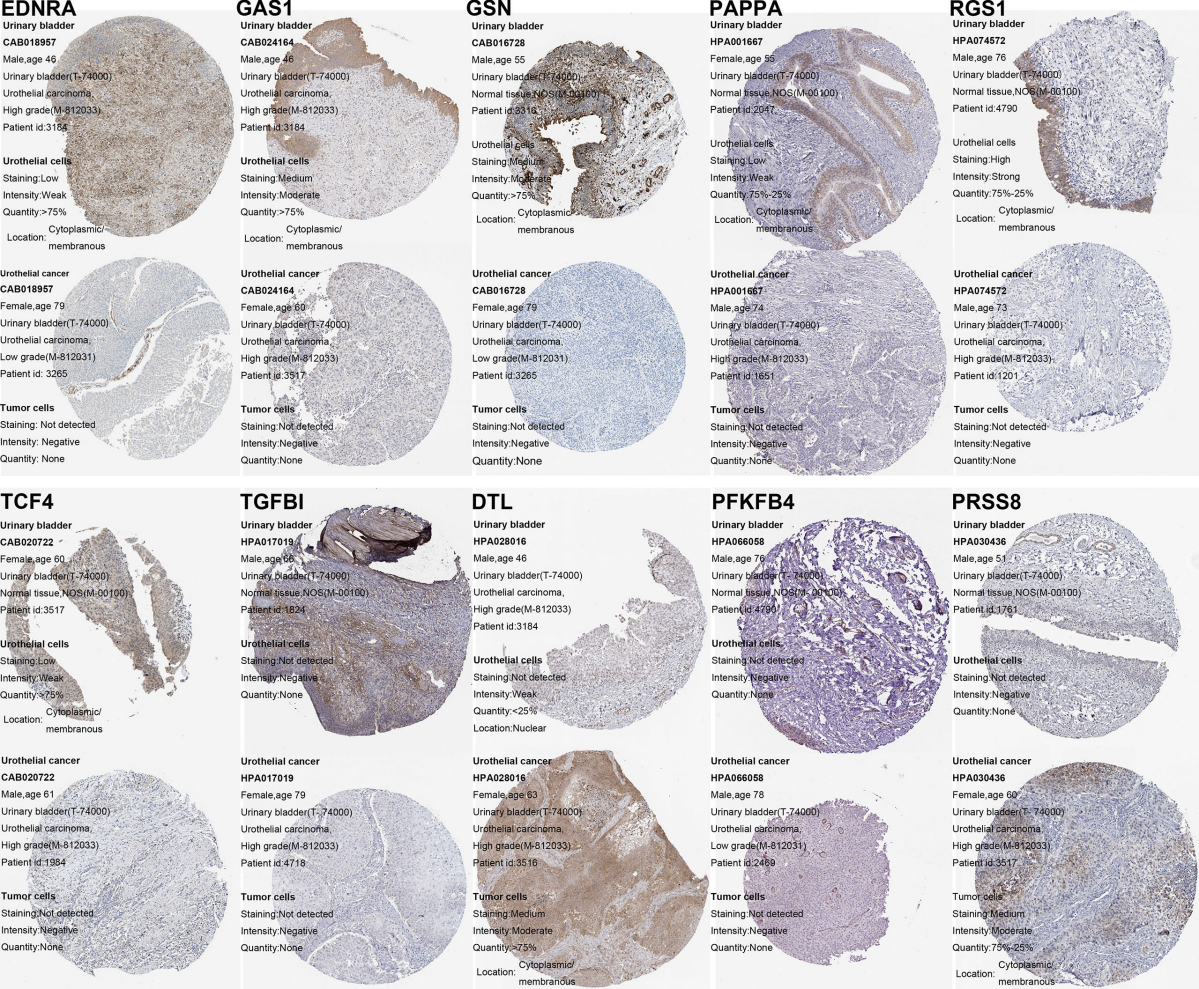
Verification of the ceRNA-associated modeled genes expression in BCa and normal bladder tissue using the HPA database.

By using the Lasso-penalized Cox regression method, 10 genes including PFKFB4, EDNRA, GSN, GAS1, PAPPA, DTL, TGFBI, PRSS8, RGS1, and TCF4 were screened out to construct the prognostic model based on TCGA_BLCA training datasets (**Figures 3C, D**). The risk score of each patient was calculated as follows: risk score = (0.136) × PFKFB4 expression + (0.042) × EDNRA expression + 0.169 × GSN expression +(-0.013) × GAS1 expression + (0.065) × PAPPA expression + (0.134) × DTL expression + (0.018) × TGFBI expression + (0.036) × PRSS8 expression + (−0.134) × RGS1 expression + (0.271) × TCF4 expression. Among them, two genes (i.e., RGS1 and GAS1) hazard ratios (HR) were less than 1, while the other eight have hazard ratios of more than 1 (**Supplementary File S8**). Using the HPA database, the protein expression of the ten modeled genes was analyzed. Of them, TGFBI and PFKFB4 was negative in BCa and normal tissues. The DTL and PRSS8 was relatively highly expressed; however, EDNRA, GAS1, GSN, PAPPA, RGS1, TCF4 exhibited significantly lower expression in tumors compared to normal tissue (**Figure 4**).

**Figure 5:**
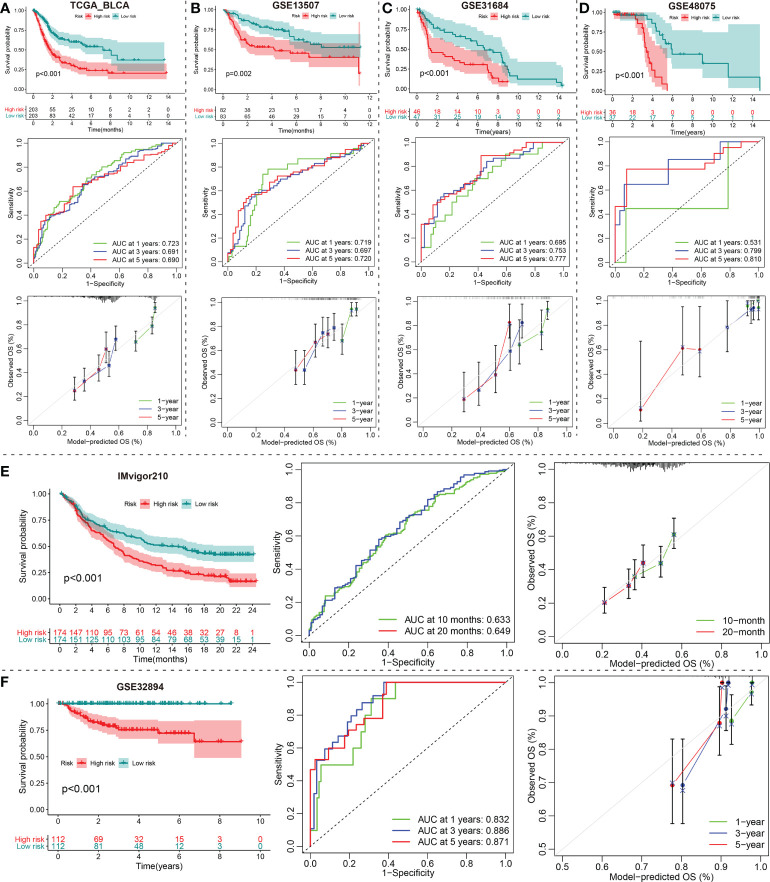
Construction and validation of a DE-mRNAs of the ceRNA network Signature. **(A)** The Kaplan-Meier, ROC and calibration curves of the 1-, 3-, and 5-year OS rate prediction for patients with BCa based on the risk score for the TCGA_BLCA training cohort. **(B–F)** Kaplan-Meier, ROC and calibration curves of the risk score for the 1-, 3-, and 5-year OS rate prediction for the testing set, including GSE13507, GSE31684, GSE48075, IMvigor210 and GSE32894. ceRNA, competing endogenous RNA; BCa, bladder cancer; DE-, differentially expressed-.

Subsequently, we stratified patients with BCa in the TCGA training set into low- and high-risk groups according to the calculated median risk score. Interestingly, Kaplan-Meier survival curve revealed that the OS in the high-risk group was clearly better than that in the low-risk group (p < 0.001; **Figure 5A**). ROC analysis suggested that the areas under curves (AUCs) employed to predict the 1-, 3-, and 5-year survival rates of patients with BCa in training set were 0.723, 0.691, and 0.690, respectively (**Figure 5A**). The calibration curves for predicting OS at 1-, 3-, and 5-year in TCGA_BLCA training group were presented in **Figure 5A**. To verify the reliability of the current prognostic prediction model, we assessed the predictive capabilities of the above 10 gene signature in other external validation dataset, GSE13507, GSE31684, GSE48075, IMvigor210 and GSE32894 dataset were downloaded from GEO database (**Figures 5B–F**). It is worth saying that the IMvigor210 cohort including 348 MIBC patients treated with Atezolizumab was adopted to further evaluate the predictive performance of the prediction models in BCa immunotherapy cohorts. The KM analyses in the validation sets yielded results consistent with those of the training set; namely, the high-risk patients were correlated with worse prognosis (**Figures 5B–F**). The ROC and calibration curves of these validation sets also demonstrated that the 10 gene signature is a good prognostic factor of BCa patients with or without ever receiving immunotherapy (**Figures 5B–F**).

### The clinical outcome predictability of the ceRNA-associated gene signature combined with clinicopathological features

**Figure 6:**
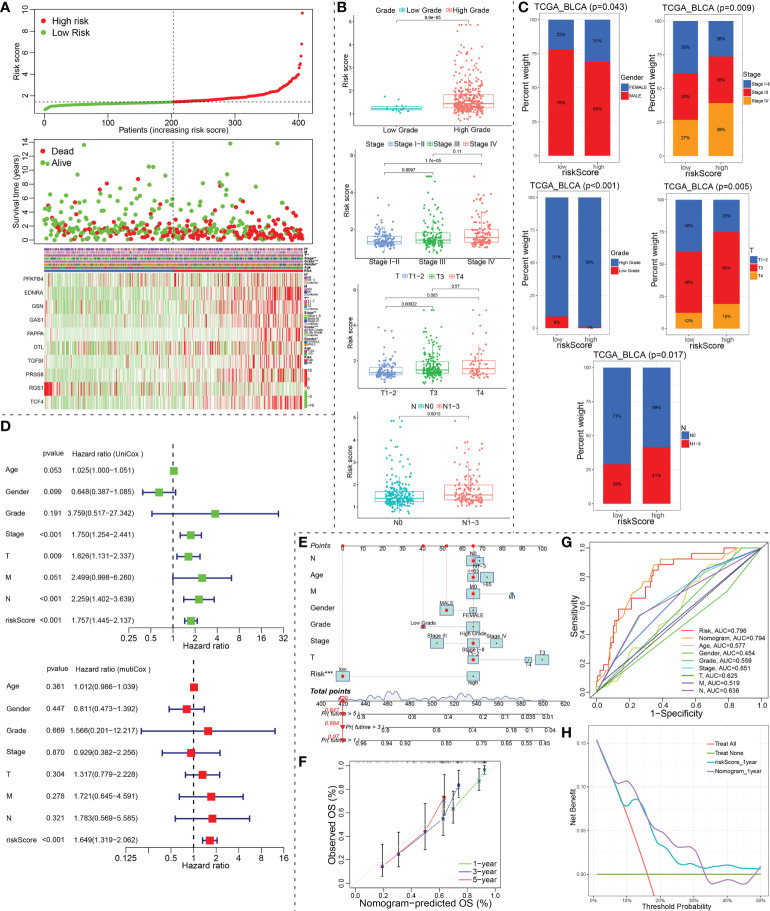
Identification of prognostic indicators for bladder cancer in training set. **(A)** The risk score curve, survival status and ceRNA-associated prognosis model gene expression heatmap were displayed from top to bottom in the TCGA_BLCA training cohort. The abscissa axis of these graphs were ranked by the risk score value. **(B, C)** Correlation between riskscore group and clinicopathological data of BCa patients *via* Wilcox rank test or Chi-square test. **(D)** The risk score was independent risk factors for bladder cancer. **(E)** Construction of a ceRNA-associated gene signature combined with clinical features nomogram. **(F)** Calibration curve of nomogram. **(G)** A multi-index ROC curve demonstrated the good discriminative abilities of the ceRNA-associated gene signature or nomograms. **(H)** DCA was applied to render clinical validity to the constructed gene signature or nomograms. ceRNA, competing endogenous RNA; BCa, bladder cancer; ROC, receiver operating characteristic; DCA, decision curve analysis. *P < 0.05, **P < 0.01 and ***P < 0.001.

In TCGA_BLCA training cohort, we evenly divided the 406 BCa patients into a low-risk group and a high-risk group according to the median risk score. The differences between the two risk groups in the distribution of risk score and survival status were further explored (**Figure 6A**). The expression heatmap showed that RGS1 showed higher expression in the low-risk group than in the high-risk group. However, the other nine modeled genes exhibited lower expression in the low-risk group than in the high-risk group (**Figure 6A**). Corresponding risk group, age, gender, grade, stage, T, N, and M were also shown in **Figure 6A**. To investigate the relationship between the ceRNA-associated gene signature and clinical features, we compared the risk scores between different clinical features using Chi-square or Wilcox nonparametric tests across the TCGA_BLCA training set. As illustrated in **Figure 6B**, patients with high grade than low grade had higher risk scores. Moreover, stage IV patients exhibited higher risk scores than stage I-II or III patients, T4 patients showed higher risk scores than T1-2 or T3 patients, and risk scores in the N1-3 was higher than in N0 (P<0.05). To confirm the clinical value of ceRNA-associated gene signature, the Chi-square test was also applied to evaluate the association between the risk score group and available clinical parameters. Obviously, there were significantly correlations between risk score group and gender, grade, stage, T, or N (**Figure 6C**). Furthermore, we conducted the univariate and multivariate Cox regression analysis revealed that the signature of the 10 ceRNA-associated genes was an independent prognostic model (univariate Cox: HR (95% CI), 1.757 (1.445-2.137); multivariate Cox: HR (95% CI), 1.649 (1.319-2.062); **Figure 6D**).

Our nomogram incorporated age, gender, grade, stage, T, N, M and risk group to predict 1-, 3-, and 5-year OS in patients with BCa (**Figure 6E**). In the nomogram, a higher total point predicts a worse prognosis. Our results exhibited that the modeled risk group had the greatest impact on the prediction of OS rate. To evaluate the calibration and discrimination of the nomogram, the Multiple ROC curve in 1-year OS and calibration curve for 1-, 3- and 5-year OS were also drawn in the TCGA_BLCA training subgroup with complete clinical data (**Figures 6F, G**). As shown in the ROC analysis, ceRNA-associated gene prognostic model (AUC, 0.796) and nomogram model (AUC, 0.794) showed a high accuracy in predicting OS. The prediction was consistent with the actual observation. The decision curve analysis (DCA) for assessment of the clinical utility was performed for the ceRNA-associated prognostic prediction model (cyan-blue line) and nomogram model (purple line) as shown in **Figure 6H**. The DCA confirmed our expectations.

### The role of ceRNA-associated gene signature in the modulation of the tumor immune microenvironment and anti-PD1/PD-L1 immunotherapy based on TCGA_BLCA training set

**Figure 7:**
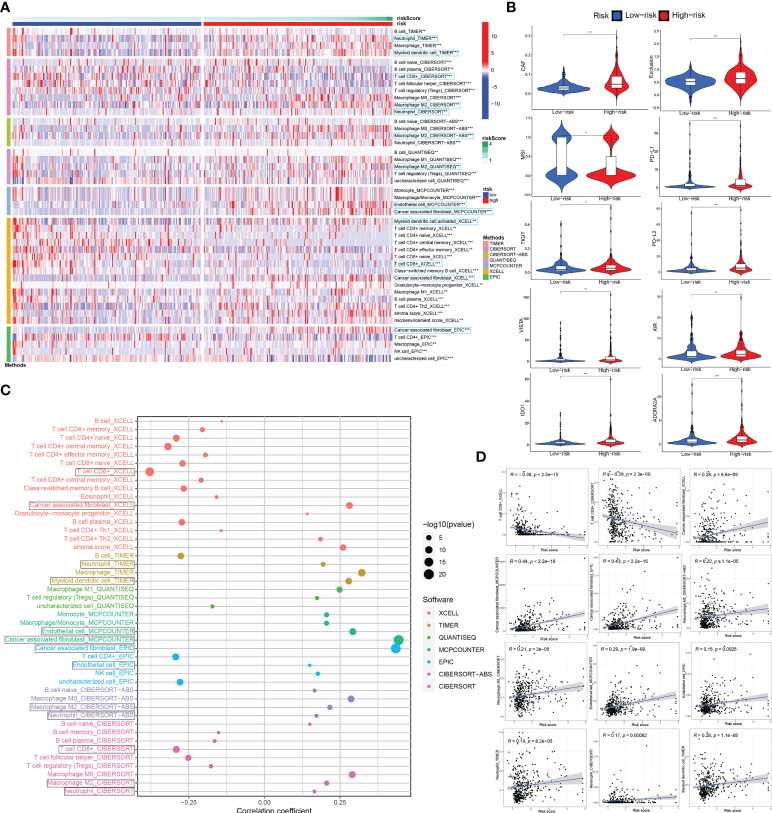
Associations between ceRNA-associated gene signature and immune-cell infiltration, immune checkpoint immunotherapies. **(A)** There were distinct differences in many immune cells infiltration between the high- and low-risk groups. **(B)** The TIDE algorithm analysis and the expression of partial immune checkpoint genes was used to predict the immune response to immune checkpoint therapy in BCa patients based on riskscore. **(C, D)** Correlation analysis between infiltrating immune cells abundance from 7 immune-infiltration algorithm and the riskscore. ceRNA, competing endogenous RNA; TIDE, tumor immune dysfunction and exclusion; BCa, bladder cancer. *P < 0.05, **P < 0.01 and ***P < 0.001.

The degree of immune cell infiltration in tumor microenvironment (TME) affects tumor occurrence, progression and therapeutic effect, especially immunotherapy. According to the 7 immune-infiltration algorithm analysis (TIMER, CIBERSORT, CIBERSORT-ABS, QUANTISEQ, MCPCOUNTER, XCELL and EPIC), a heatmap of 45 significant differences at the level of immune cells (sigDICs) in the high vs. low risk groups (Wilcoxon test, P < 0.01) is presented as **Figure 7A**, and these differential immune cells including CD8+ T cells, cancer associated fibroblast, macrophage M2, neutrophils, myeloid dendritic cell and endothelial cell, etc. Meanwhile, we performed the spearman’s correlation analysis (P < 0.01) between riskscore and immune-infiltration cells (**Figures 7C, D**). To predict the response of the two risk groups to immunotherapy, the tumor immune dysfunction and exclusion (TIDE) algorithm analysis and partial immune checkpoint gene expression was used to predict the immune checkpoint therapy response based on riskscore group in the TCGA_BLCA training cohort (**Figure 7B**).

### The investigation of ceRNA-associated gene signature in the tumor immune microenvironment and immunotherapy based on IMvigor210 and GSE32894 external test sets

**Figure 8:**
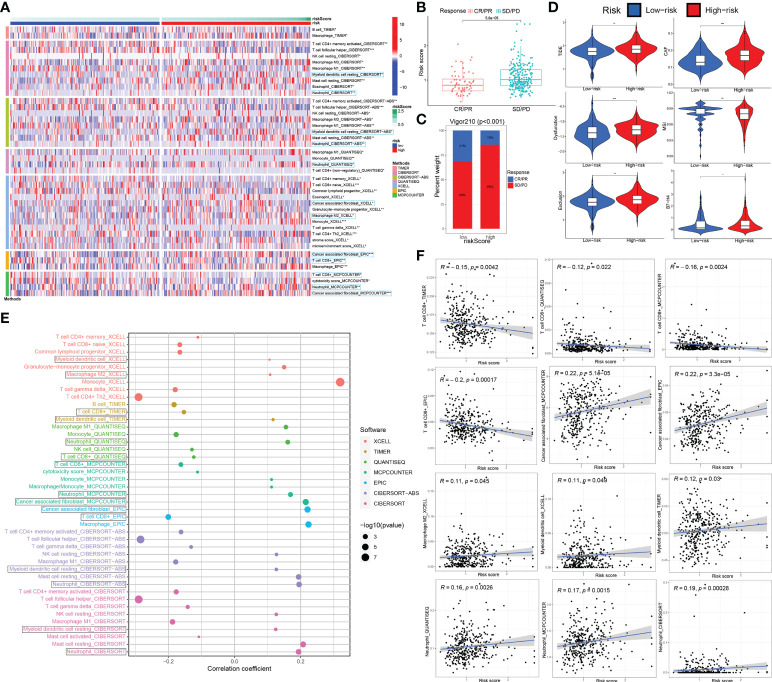
The ceRNA-associated gene signature is an important indicator of immune cell infiltration and immunotherapeutic effect. **(A)** The immune cell infiltration difference from 7 immune-infiltration algorithm in high-risk groups compared to low-risk groups. **(B)** The association between the risk score and immunotherapy outcome by wilcox test. **(C)** Correlations among the riskscore group and immunotherapy outcome variables. **(D)** Using TIDE algorithm analysis and the expression of partial immune checkpoint genes, immune checkpoint blockade therapy response based on riskscore was predicted. **(E, F)** Spearman association analysis between riskscore and immune cell infiltration was implemented. ceRNA, competing endogenous RNA; TIDE, tumor immune dysfunction and exclusion. *P < 0.05, **P < 0.01 and ***P < 0.001.

Immune cell infiltration in tumor microenvironment (TME) can reflect the immune response to tumor. Analogously, according to the 7 immune-infiltration algorithm analysis for IMvigor210 and GSE32894 datasets, a heatmap showed the sigDICs between high- and low-risk groups (Wilcoxon test, P < 0.05), and these differential immune cells were very similar to sigDICs from the TCGA_BLCA training set (**Figure 8A** **;** **Supplementary Figure 2A**). Furthermore, Spearman’s rank correlation analysis between riskscore and infiltration abundances of immune cells was also implemented (**Figure 8E** **;** **Supplementary Figure 2E**); and the linear fits between riskscore and the concerned immune cells commonly affecting the efficacy of immunotherapy were further plotted (**Figure 8F** **;** **Supplementary Figure 2F**). Afterward, we used TIDE and partial immune checkpoint gene expression to evaluate the potential clinical efficacy of immune checkpoint therapy based on riskscore group in IMvigor210 and GSE32894 cohorts (**Figure 8D** **;** **Supplementary Figure 2D**).

To further validate the correlation of riskscore group with immunotherapy response, we analyzed 348 MIBC cases in IMvigor210 that received Atezolizumab immunotherapy by Chi-square and Wilcox tests (**Figures 8B, C**). We observed that the riskscore of patients with SD/PD was significantly higher than that with CR/PR (p < 0.0001; Wilcox test). We showed that rate of clinical immunotherapy SD/PD was distinctly higher in the high- vs. low-risk groups (p < 0.0001; chi-square test). Additionally, we explored the relationship between risk scores and clinical characteristics of BCa patients in the GSE32894 dataset. There were statistically significant differences between G3/G4 and G1/G2 grade tumors in high vs. low-risk group (p < 0.001 by Wilcox and chi-square tests). Similarly, statistically significant differences between T2-4 and Ta-1 groups (p < 0.001) are also indicated in **Supplementary Figures 2B, C**.

### Exploration of the downstream molecular mechanism and chemosensitivity based on ceRNA-associated gene model

**Figure 9:**
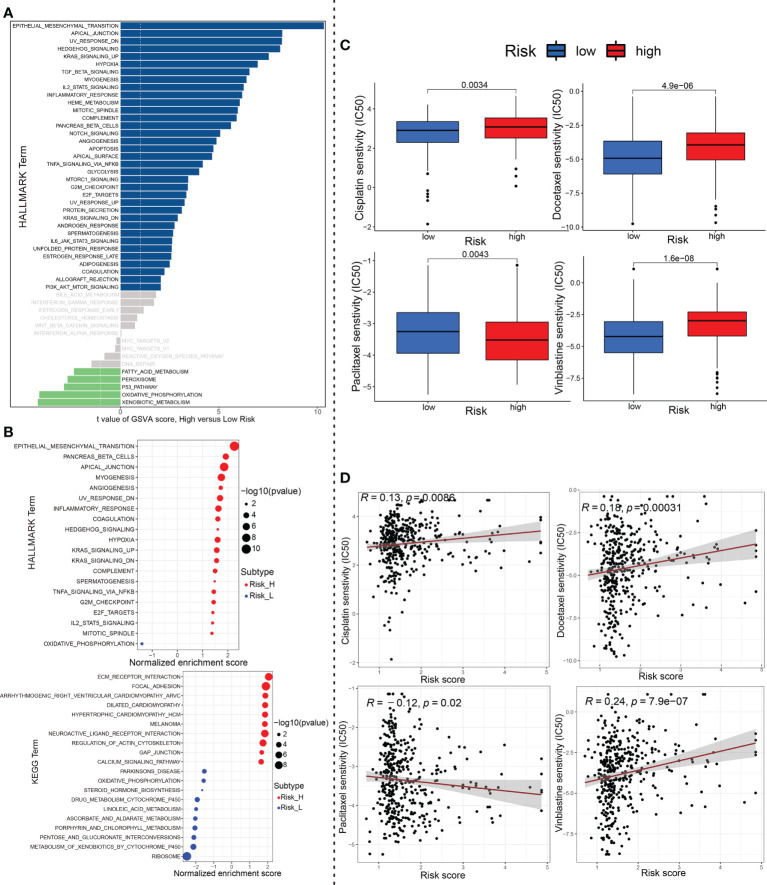
The underlying molecular mechanisms and chemotherapeutics. **(A)** Differences in pathway activities scored by GSVA of HALLMARK gene sets between high-risk and low-risk group. Navy blue, significantly upregulated enrichment; green, significantly downregulated enrichment; grey, no enrichment. **(B)** Most significant enriched 20 GSEA pathways in high- vs. low-risk group were displayed separately based on KEGG and HALLMARK gene sets. Red, significant positive enrichment; green, significant negative enrichment. **(C)** Wilcox group analysis and **(D)** spearman correlation analysis indicated that the ceRNA-associated gene model is robust to drug sensitivity of Cisplatin, Docetaxel, Paclitaxel and Vinblastine. GSVA, Geneset variation analysis; GSEA, gene set enrichment analysis; ceRNA, competing endogenous RNA.

To explore the underlying molecular mechanism of ceRNA-associated gene model, we next performed GSVA of Hallmark gene sets to figure out dynamics of biological processes and pathways between the high- and low-risk groups based on TCGA_BLCA training dataset. As shown in **Figure 9A**, 35 Hallmark pathways (such as “Epithelial Mesenchymal Transition”) were significantly upregulated, while 5 Hallmark pathways were significantly downregulated, in the high-risk group compared to the low-risk group (p-value < 0.05; using R limma). Of these, the pathway with the most significant changes was the “Epithelial Mesenchymal Transition”. Afterwards, we conducted gene-set enrichment analysis (GSEA), based on HALLMARK and KEGG gene sets. Among them, the most significantly enriched 20 HALLMARK (For instance, ‘Epithelial Mesenchymal Transition’, ‘Inflammatory Response’, ‘Angiogenesis’, ‘Hypoxia’, ‘Hedgehog_Signaling’ in the high-risk group, etc.) or KEGG pathways (e.g., ‘Ecm Receptor Interaction’, ‘Focal Adhesion’, ‘Regulation Of Actin Cytoskeleton’, ‘Gap Junction’, ‘Pathways_In_Cancer’ in the high-risk group, etc.) are shown in **Figure 9B**.

Finally, we analyzed the IC50 of four chemotherapeutic drugs (Cisplatin, Docetaxel, Paclitaxel and Vinblastine) commonly used to treat BCa through the pRRophetic algorithm. Compared with the effect of low-risk group on Cisplatin, Docetaxel and Vinblastine sensitivity, the effect of high-risk group on these drug sensitivity was relatively weak. Conversely, Paclitaxel showed opposite results (P<0.01, wilcox test; **Figure 9C**). In addition to this, we further performed the spearman correlation between the risk score with the IC50 of above these chemotherapy drugs. As evident from **Figure 9D**, the results of correlation analysis (P<0.05, spearman correlation test) were consistent with those of aforementioned riskscore group wilcox analysis.

### The ceRNA-associated partial circRNAs expression validation by qRT-PCR; the biological function of hsa_circ_0067900 *in vitro*

**Figure 10:**
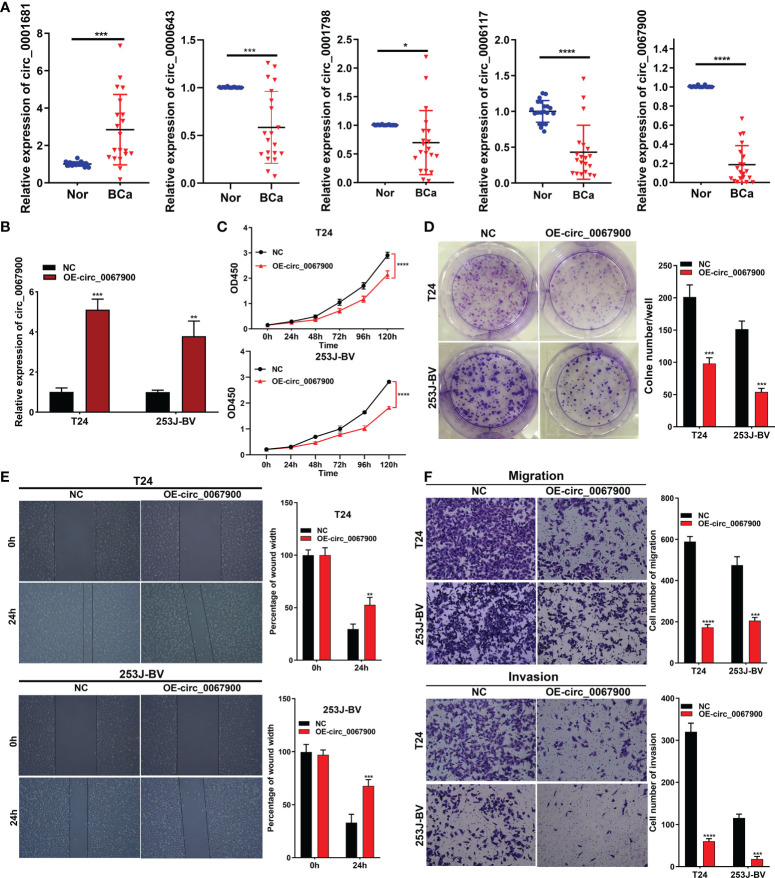
circ_0067900 overexpression exerted a tumor suppressive effect in BCa cells. **(A)** Five circRNAs were randomly chosen to verify the circRNA-sequencing results in 20 pairs of samples by quantitative real-time PCR. **(B)** Lentiviral transduction efficiencies were evaluated using RT-qPCR in T24 and 253J-BV cells. **(C)** CCK-8 proliferation assay. **(D)** OE-circ_0067900 inhibited monoclonal formation ability of BCa cells. **(E)** Scratch assay of T24 and 253J-BV cells. Experiments were terminated after scratch for 24 h. **(F)** Migration and invasion were evaluated using a transwell assay without and with Matrigel, respectively. Data are showed as means ± SD; n = 3. SD, standard deviation; OE-, Overexpression; NC, negative control vector; CCK-8, cell counting kit-8; OD, optical density. *P < 0.05, **P < 0.01, ***P < 0.001 and ****P < 0.0001.

The relative expression of randomly selected five circRNAs (circ_0001681, circ_0000643, circ_0001798, circ_0006117 and circ_0067900) were validated in 20 pairs of BCa and paracancerous tissues (P < 0.05, wilcox test), and these findings were completely in accord with the data obtained from the our sequencing analysis (**Figure 10A**). Sanger sequencing validated the sequence on the junction sites of circ_0067900 (**Supplementary Figure 3A**). Additionally, we conducted RNase-R + digestion on the circ_0067900 and examined the resistance of circ_0067900 to RNase-R+ digestion by RT-qPCR. The results indicated that the circ_0067900 showed varying degrees of resistance to RNase-R+ digestion compared to corresponding linear mRNAs (**Supplementary Figure 3B**).

Additionally, to explore the effects of the most prominent downregulated circ_0067900 on the biological behavior of BCa cells. Lentiviral vector expressing GFP (negative control, NC) or GFP and circ_0067900 (OE-circ_0067900) was used to transfect in T24 and 253J-BV cells. Fluorescence microscopy showed strong green fluorescence in the majority of cells at 72h postinfection, signifying a high efficiency of lentiviral transduction and infection. And, transfection efficiency was confirmed with real-time PCR (**Figure 10B**). The CCK-8 and clonogenic formation experiments results revealed that the OE-circ_0067900 markedly weakened the proliferation ability of T24 and 253J-BV cell lines compared with NC (**Figures 10C, D**). Then, we conducted the effects of circ_0067900 on BC cells migration and invasion capacity. The scratch wound healing assay results reflected that circ_0067900 overexpression significantly impaired the migration of BCa cells compared with NC group (**Figure 10E**). Consistently, transwell assay with or without Matrigel also showed that OE-circ_0067900 dramatically suppressed the migration and invasion of T24 and 253J-BV cell lines (**Figure 10F**). Overall, these results indicated that OE-circ_0067900 played a crucial function in BCa cell proliferation, migration, and invasion.

## Discussion

Bladder cancer (BCa), one of the most common urogenital tumors, has high incidence, recurrence, and mortality (2). Accumulating evidence has showed that dysregulated circRNA expression affects a variety of biological functions, including cell proliferation, apoptosis, invasion, migration, sensitivity and immune cell infiltration in BCa. For instance, Zhang et al. demonstrated that hsa_circ_0007813 was increased in BCa, and it can competitively binds hsa-miR-361-3p to modulate IGF2R expression, thereby inhibiting tumor migration, invasion, and proliferation as well as autophagy (30). Wei et al. revealed that hsa_circ_0008399 inducing by EIF4A3 inhibited apoptosis, bound WTAP to favor formation of the WTAP/METTL3/METTL14 m6A methyltransferase complex and boosted expression of TNFAIP3 *via* enhancing its mRNA stability in an m6A-dependent way, and attenuated BCa chemosensitivity to cisplatin (31). It has been reported that CircZNF609 is upregulated markedly in BCa, boosts cell progression and cisplatin chemoresistance by novel miR-1200/CDC25B path, and serve as a novel prognostic predictor and therapeutic target in BCa (32).

Recently, many studies about the biological functions of circRNA in regulating tumor-infiltrating immune cells, including CD8+ T cell (33, 34), cancer-associated fibroblasts (14), M2/M1 macrophages (35, 36), and Treg cells (24), etc, have been investigated in diverse malignancies. Immunotherapy in BCa has shown promising results, but how to better figure out the heterogeneity of BCa is still a highly debated topic.

Taken together, circRNAs act as hidden biomarkers for diagnosis and prognosis, and therapeutic targets in patients with BCa. Prior studies have identified the role of circRNAs in BCa, but ignored the global expression profile and accurate molecular mechanism of BCa-specific circRNAs. In the present study, we explored the expression profiles of circRNAs, composed a ceRNA network, constructed a ceRNA-associated prognostic signature and analyzed the correlation between the model and clinical traits, the commonly used chemotherapeutic agents sensitivities, the tumor-infiltrating immune cell and immunotherapy effects based on multiple large-sample datasets in BCa using bioinformatics and statistical tools.

In this study, *via* high-throughput RNA sequencing (RNA-seq), we here explored the expression profiles of circRNAs and determined 87 significantly DEcircRNAs, in five BCa samples compared to paired noncancerous peritumoral samples. Of note, our previous studies (16, 17) confirmed that the circ_0026782, circ_0004826, circ_0077837, circ_0001946, and circ_0030586 were significantly downregulated, whereas circ_0003141 and circ_0008039 was dramatically upregulated in BCa vs. paired non-tumorous tissues, which were consistent with our circRNA sequencing data; besides, circ_0077837, circ_0004826 and circ_0030586 served as tumor suppressors and prognosis predictors in BCa. Additionally, some circRNA among our sequencing such as circ_0008039, circ_0000745, circ_000074, circ_0077837, circ_0030586, and circ_0001946, etc, were also closely associated with malignant biological behavior of other cancer types (37–42).

In this study, to validate the accuracy of circRNA-sequencing, five DE-circRNAs (including circ_0001681, circ_0000643, circ_0001798, circ_0006117 and circ_0067900), were randomly selected and detected by qRT-PCR in 20 pairs of BCa and matched adjacent tissues, and the results confirmed the RNA-seq data. Of them, we investigated the biological roles by overexpressing circ_0067900 among T24 and 253J-BV cells. CCK-8 and colony-formation assays revealed that the OE-circ_0067900 significantly restrained BCa cells proliferation. Moreover, we examined the impacts of OE-circ_0067900 on BCa cells migration and invasion. Transwell and wound healing assays exhibited that OE-circ_0067900 remarkably attenuated BCa cells migration and invasion capacity. Therefore, these data demonstrated that circ_0067900 could act as a tumor suppressor in BCa development and progression.

Subsequently, we downloaded three miRNA (GSE11224, GSE113786 and GSE113740) and two mRNA (GSE13507 and GSE37815) microarray data from GEO database, and identified 178 intersection DEmiRNAs (including 60 upregulated and 118 downregulated) and 326 intersection DEmRNAs (56 upregulated and 270 downregulated) in BCa vs. adjacent normal tissues. To identify the DEcircRNAs-DEmiRNAs-DEmRNAs regulatory relationships, we performed the prediction of circRNA–miRNA and miRNA–mRNA interactions based on the popular online tools, and then constructed a ceRNA network following the “ceRNA network hypothesis”. In this ceRNA network, previous studies have confirmed that miR-3194-3p/AQP1 (43), miR-1246/AXIN2 (44), miR-326/E2F2 (45), miR-326/CDCA5 (46), and miR-17-3p/RGS2 (47), etc, interaction relations play important roles in the occurrence and development of various cancers. Notably, these researches also to some extent reflect the veracity of the predicted ceRNA network in the current study.

To better clarify the potential functional effects for the 87 DE-circRNAs in BCa, the 152 DEmRNAs presented in the ceRNA network were used for the GO annotation and KEGG pathway analyses. As showed, the significant GO terms were ‘regulation of epithelial to mesenchymal transition (GO:0010717)’, ‘regulation of mitotic cell cycle phase transition (GO:1901990)’, ‘regulation of Wnt signaling pathway (GO:0030111)’, ‘actin binding (GO:0003779)’, and ‘focal adhesion (GO:0005925)’, etc. Furthermore, the current study illustrated that several tumor-associated pathways were remarkedly enriched, including ‘MicroRNAs in cancer’, ‘PI3K−Akt signaling pathway’, ‘Focal adhesion’, ‘Ras signaling pathway’ and ‘MAPK signaling pathway’. Previous researches have demonstrated that the activations of these above biological processes promote BCa cell proliferation, invasion and metastasis (48–51). Tan et al. reported that circST6GALNAC6, modulated by the transcription factor SP1, inhibits BCa metastasis through binding miR-200a-3p to regulate the STMN1/EMT axis (48), and Su et al. revealed that Circ_0000658 enhanced BCa cell proliferative, migratory and invasive capacities by miR-498/HMGA2/EMT signaling axis (49). As reported, circCEP128 promotes BCa progression through activating MAKP signaling pathway *via* miR-145-5p/MYD88 path (50). Therefore, it is reasonable to assume that these dysregulated circRNAs in BCa act principally *via* regulating the epithelial to mesenchymal transition, cell cycle and the aforementioned pivotal pathways, which then affect tumor cell aggression.

Afterward, we constructed a ceRNA-associated 10-gene signature to explore the relationship between BCa and circRNA-related ceRNA network based on the TCGA cohort using Lasso-penalized Cox regression algorithm. Among these selected genes, the protein expression of them was confirmed by the HPA database. And, increasing evidence has indicated that some of them may play different and crucial biological functions in the progression of cancer. As discovered by Luo et al. (52), DTL overexpression was associated with malignant biological behavior and unfavorable prognosis of BCa patients, and furthers BCa progression through inducing EMT *via* the AKT/mTOR pathway. It has been revealed that PFKFB4 is highly expressed in many types of human tumors, including breast, prostate, and bladder cancers (53–55), indicating that PFKFB4 plays an essential role in tumor development and/or progression. Huang et al. suggested that RGS1 is upregulated by IFN/STAT1 path, and weaken trafficking of circulating T cells to tumors by suppressing calcium influx and inhibiting activation of the ERK and AKT kinases; conversely, its knockdown in adoptively transferred tumor-specific CTLs remarkedly increase their infiltration and productively suppress tumor growth in lung and breast tumor grafts *in vivo*, which is further improved when combined with PD-L1 checkpoint inhibition (56); and, RGS1 is a novel marker and promoting factor for CD8+ T-cell exhaustion (57). AS indicated by Han et al. (58), RGS1 and RGS13 silencing act together to enhance CXC chemokine receptor signaling in human germinal center B lymphocytes. It has been revealed that GAS1, was directly modulated by the transcription factor FOXM1, impairs cell invasion, proliferation and aerobic glycolysis of colorectal cancer both *in vitro* and *in vivo*, through inhibiting EMT and the Warburg effect by AMPK/mTOR/p70S6K axis (59); similarly, GAS1 acts as a tumor suppressor in gastric and breast cancers, etc (60, 61).

Notably, the signature was validated in several external BCa datasets (GSE13507, GSE31684, GSE48075, IMvigor210 and GSE32894). Obviously, patients in the low-risk group enjoyed a favorable survival advantage by using Kaplan–Meier analysis. Surprisingly, the each AUC values of ROC plots in 1, 3 and 5 years were presented in **Figure 5**, and nearly all high, in the TCGA_BLCA training dataset and the five testing datasets, which illustrated that this model had excellent accuracy. Subsequently, we found that the modeled riskscore was significantly correlated with gender, grade, stage, T, and N. Furthermore, BCa patients with stage III/IV, high-grade, T3/4, N1-3 or the immunotherapy ineffective-response indicated remarkably higher riskscores than those with oppositional characteristics based on wilcox-test. Of course, according to chi-square test, the risk group ratio of BCa patients with different gender, grade, stage, T, N or immunotherapy response also presented a significant difference that was consistent with above wilcox-test results. Therefore, a nomogram integrating these features was established that could predict clinical survival outcomes well.

The results of 7 immune-infiltration algorithm analyses, indicated that the risk score exhibited a significant positive association with the infiltration of cancer-associated fibroblasts (CAFs), macrophage M2, neutrophils, myeloid dendritic cell and endothelial cell, etc; however, risk score was dramatically and negatively correlated with the infiltration level of CD8+ T cells. In a previous study, CAFs, which are the activated phenotype of fibroblasts and constitute the most abundant and heterogeneous stromal cells in the TME, are crucially implicated in BCa development, progression, chemoresistance and anti-PD1/PD-L1 immunotherapy response (62–64). Martínez et al. reported that BMP4, produced by BCa cells, induces M2 polarization of the monocytes/macrophages, contributing to the production of cytokines that favor tumor invasion partly through modulating miR-21/BMPR2 axis (65). Kobatake et al. revealed that Kdm6a-deletion activates cytokine and chemokine pathways, leads to M2 Macrophage Polarization, and increases BCa development in cooperation with p53 dysfunction; notably, dual inhibition of IL6 and chemokine (C-C motif) ligand 2 effectively reversed the process (66). Moreover, high macrophage M2 infiltration and low CD8 T cells in the high-risk group of BCa were priorly demonstrated to be correlated with destitute response in immunotherapy (67). In BCa, exosome-originated circTRPS1 from tumor cells can regulate CD8+ T cell exhaustion and the balance of intracellular reactive oxygen species through the miR141-3p/GLS1 axis (33). Through single-cell RNA sequencing, Chen et al. also illustrated that monocyte/macrophages polarization toward M2 phenotype, LAMP3 + DC subgroup recruiting regulatory T cells, and inflammatory cancer-associated fibroblasts (iCAFs) in the tumor region, all potentially involve in the formation of an immune-suppressive TME and tumor progression, which is significantly associated with poor prognosis of BCa patients (68).

After analyzing differently expressed genes in high- vs. low-risk groups, GSVA results based on Hallmark gene sets revealed that the pathways of ‘Epithelial Mesenchymal Transition’, ‘Inflammatory Response’, ‘Angiogenesis’, ‘Hypoxia’, ‘Hedgehog Signaling’, ‘Kras Signaling’, ‘G2M Checkpoint’, ‘Tnfa Signaling *Via* Nfkb’ and ‘IL2 Stat5 Signaling’ were enriched in the high-risk group. Similarly, according to KEGG pathways, The GSEA results also showed that ‘Ecm Receptor Interaction’, ‘Focal Adhesion’, ‘Regulation Of Actin Cytoskeleton’, ‘Gap Junction’, ‘Pathways In Cancer’, were significantly enriched in the high-risk group. These results illustrated that ceRNA-related 10-gene signature in BCa may be correlated with aforementioned pathways. Previous studies have suggested that the above enriched pathways, such as ‘Epithelial Mesenchymal Transition’, ‘Inflammatory Response’, ‘Angiogenesis’, and ‘Hypoxia’ pathways, etc, were closely connected to tumor cell the occurrence, development, invasion, metastasis, and the regulation of the tumor microenvironment in BCa (48, 69–71).

Immune checkpoint blockades (ICBs), including antibodies to PD-1, PD-L1 or CTLA-4, etc, may be effective in treating BCa (72), nevertheless, their response rates remain relatively low in unselected tumor patients (73–75). ICBs serve as an emerging class of biologics that interact with the immune system to excite an antitumor response *via* immune cells (76). Recent research showed that efficacy of immune checkpoint inhibitors may be associated with multiple factors, such as TIME regulation, immune checkpoint expression, and the process of T cell dysfunction and exclusion during the immune cycle (77). The TIDE is developed by Jiang et al. (78) to predict the likelihood of immunotherapy responsiveness based on modeling the mechanisms of tumor immune evasion. Of note, our study revealed that a unique immune landscape, immune checkpoint genes expression and immunotherapeutic responses was exhibited between high- and low-risk prognostic groups; thus, the integrative analysis speculated that the immunotherapy effect of the BCa patient subgroup with high-risk may be advanced. Besides, we also investigated the sensitivity to chemotherapy agents usually used for treating BCa *via* calculating IC50 value and select out candidate small-molecule compounds. Collectively, these findings may offer suitable therapy alternatives for BCa patients. These results underscore the importance of the circRNA-related ceRNA networks in BCa progression by modulating TIME or immune checkpoint genes. Thus, targeting the hub molecules in the ceRNA network or TIME (specifically CAFs or M2 Macrophage), can be exploited as a cancer vulnerability in the progression of BCa and should be strongly considered as a reliable therapeutic strategy.

## Conclusion

In conclusion, our research indicated that circRNA-associated gene signature is highly related to BCa. Moreover, the risk score or group based on the signature arose from 10 circRNA-related genes appraised this original model through multiple clinical scenarios such as clinicopathological features, tumor immune microenvironment, and chemotherapy. Additionally, the results also found that has_circ_0067900 may act as a tumor suppressor, and promising biomarker for diagnostic and prognostic predication in BCa patients.

## Data availability statement

The original contributions presented in the study are included in the article/**Supplementary Material**. Further inquiries can be directed to the corresponding author.

## Ethics statement

Written informed consent was obtained from the individual(s) for the publication of any potentially identifiable images or data included in this article.

## Author contributions

HH, CS, and ZL conceived and designed the study. CS, YLZ, and LD performed the experiments and analyzed the results. ZZ, ZLW, and SY collected the clinical specimens. CS and ZL wrote the manuscript. ZJW, YZ, YQ, GZ, YL, MZ and SH edited the manuscript and provided critical comments. All authors contributed to the article and approved the submitted version.

## Funding

This work was supported by the grants from the Youth Fund of Tianjin Medical University Second Hospital (No. 2020ydey09), the Natural Science Foundation Project of Tianjin (No. 18PTLCSY00010 and No. 20JCQNJC00550), Tianjin Municipal Health Industry Key Project (No. TJWJ2022XK014) and the Tianjin Urological Key Laboratory Foundation (No. 2017ZDSYS13).

## Acknowledgments

We sincerely thank all participants in the study.

## Conflict of interest

The authors declare that the research was conducted in the absence of any commercial or financial relationships that could be construed as a potential conflict of interest.

## Publisher’s note

All claims expressed in this article are solely those of the authors and do not necessarily represent those of their affiliated organizations, or those of the publisher, the editors and the reviewers. Any product that may be evaluated in this article, or claim that may be made by its manufacturer, is not guaranteed or endorsed by the publisher.

## Supplementary material

Supplementary Table 1 The primers used for real-time PCR are designed and synthesized by Sango Biotech (Shanghai, China) as well as shown in Table 1.

Supplementary Table 2 The association analysis between expression of the filtered 12 miRNAs and clinicopathological factors of patients with BCa. Exp., Expression level; Lymphatic met., Lymphatic metastasis. ^*^P<0.05; ^**^P<0.01; ^***^P<0.001; Fisher’s exact test.

Supplementary Figure 1 A correlation between 12 DE-miRNAs and 115 DE-mRNAs form TCGA dataset. **(A)** The Sankey diagram was plotted according to the correspondence between 12 DE-miRNAs and 115 DE-mRNAs. **(B)** A correlation analysis of 12 DE-miRNAs and 115 DE-mRNAs expression data. Red lines indicate positive co-expression relationships while blue lines indicate negative co-expression relationships. miRNA, microRNA; differentially expressed-, DE-.

Supplementary Figure 2 We carried out an immune cell infiltration analysis and the efficacy prediction of immunotherapy based on ceRNA-associated gene signature in the test GSE32894 datasets. **(A)** Heat-mapping demonstrated many significant differences in immune cell infiltration between the high- and low-risk groups. **(B, C)** Wilcox and chi-square tests were used to assess the association between the ceRNA-associated gene risk signature and clinicopathological features, including the depth of tumor invasion and tumor grade. **(D)** Based on riskscore, the TIDE algorithm and the expression of immune checkpoint genes were then employed to potentially assess responses to immune checkpoint inhibitor. **(E, F)** The Spearman correlation between tumor-infiltrating immune cells and riskscore.

Supplementary File S1 Clinical, histopathological and survival information of TCGA_BLCA and multiple GEO datasets.

Supplementary File S2 The DE-circRNAs for 5 paired BCa tissues versus adjacent normal tissues.

Supplementary File S3 The DE-miRNAs in BCa tissues vs. Controls from GSE11224, GSE113786 or GSE113740 datasets.

Supplementary File S4 The DE-mRNAs in BCa tissues vs. Controls from GSE13507 and GSE37815 datasets.

Supplementary File S5 Predicted miRNA binding sites in these DE-circRNAs.

Supplementary File S6 The GO Enriched terms for DE-mRNAs in the ceRNA network.

Supplementary File S7 The KEGG pathway analysis for DE-mRNAs in the ceRNA network.

Supplementary File S8 The ceRNA-associated gene model parameters (i.e., Coef, HR (95% CI), Pvalue).
