# Supplementary material for: Identification of a dysregulated CircRNA-associated gene signature for predicting prognosis, immune landscape, and drug candidates in bladder cancer
Source: Front Oncol. 2022 Oct 10;12:1018285. doi: 10.3389/fonc.2022.1018285 (PMC9589509; doi:10.3389/fonc.2022.1018285)
Supplement: Supplementary Table 1 — The primers used for real-time PCR are designed and synthesized by Sango Biotech (Shanghai, China) as well as shown in Table 1. [file Table_1.docx]

**Supplementary Table1,** The primers used for real-time PCR are designed and synthesized by Sango Biotech (Shanghai, China) as well as shown in Table 1.

| **Gene Name** | **Primer Type** | **Primer Sequence** | **Product Length** |
| --- | --- | --- | --- |
| hsa_circ_0001681 | Forward primer | 5’-TGCGTCTAACATCTGCGACTC-3’ | 182 |
|  | Reverse primer | 5’-GCTCCTCCTGTAAAACCCCT-3’ |  |
| hsa_circ_0000643 | Forward primer | 5’-TTACCAGTTCAATGCACAGATGG-3’ | 190 |
|  | Reverse primer | 5’-GTTGCACACCCTGCACATC-3’ |  |
| hsa_circ_0001798 | Forward primer | 5’-CCAAAAGACACTTTCAGCCAAGT-3’ | 154 |
|  | Reverse primer | 5’-TCCATAATCTGTCTTGCATCCGA-3’ |  |
| hsa_circ_0006117 | Forward primer | 5’-GACCACAAGAACAGCAAGCA-3’ | 174 |
|  | Reverse primer | 5’-ACCAGGAATCCATGCTTATCTG-3’ |  |
| hsa_circ_0067900 | Forward primer | 5’-GATAGAACCCCACCACCACC-3’ | 168 |
|  | Reverse primer | 5’-GGAAACTTTGGAAGGTGGTGA-3’ |  |
| PHC3 | Forward primer | 5’-ACAGCAGTCAAGTATGTCCCA-3’ | 156 |
|  | Reverse primer | 5’-CTTGCCGTTAGGGTAGGGG-3’ |  |
| GAPDH | Forward primer | 5’-CGGAGTCAACGGATTTGGTC-3’ | 180 |
|  | Reverse primer | 5’-TTCCCGTTCTCAGCCTTGAC-3’ |  |
