# Supplementary material for: Identification of a dysregulated CircRNA-associated gene signature for predicting prognosis, immune landscape, and drug candidates in bladder cancer
Source: Front Oncol. 2022 Oct 10;12:1018285. doi: 10.3389/fonc.2022.1018285 (PMC9589509; doi:10.3389/fonc.2022.1018285)
Supplement: Supplementary Table 2 — The association analysis between expression of the filtered 12 miRNAs and clinicopathological factors of patients with BCa. Exp., Expression level; Lymphatic met., Lymphatic metastasis. *P<0.05; **P<0.01; ***P<0.001; Fisher’s exact test. [file Table_2.docx]

**Supplementary Table2,** The association analysis between expression of the filtered 12 miRNAs and clinicopathological factors of patients with BCa.

| **Variables** | **miR-17-3p**  **Exp.** | | **miR-191-5p**  **Exp.** | | **miR-26b-3p**  **Exp.** | | **miR-29b-1-5p Exp.** | | **miR-3173-5p Exp.** | | **miR-326**  **Exp.** | |
| --- | --- | --- | --- | --- | --- | --- | --- | --- | --- | --- | --- | --- |
|  | Low  (n=205) | High  (n=204) | Low  (n=205) | High  (n=204) | Low  (n=205) | High  (n=204) | Low  (n=205) | High  (n=204) | Low  (n=205) | High  (n=204) | Low  (n=205) | High  (n=204) |
| **Gender** |  |  |  |  |  |  |  |  |  |  |  |  |
| Male (n=302) | 146 | 156 | 143 | 159 | 138 | 164**^**^** | 150 | 152 | 159 | 143 | 148 | 154 |
| Female (n=107) | 59 | 48 | 62 | 45 | 67 | 40 | 55 | 52 | 46 | 61 | 57 | 50 |
| **Age** |  |  |  |  |  |  |  |  |  |  |  |  |
| ≤65 (n=162) | 80 | 82 | 77 | 85 | 71 | 91**^*^** | 71 | 91**^*^** | 87 | 75 | 86 | 76 |
| >65 (n=247) | 125 | 122 | 128 | 119 | 134 | 113 | 134 | 113 | 118 | 129 | 119 | 128 |
| **Stage** |  |  |  |  |  |  |  |  |  |  |  |  |
| I-II (n=133) | 56 | 77**^*^** | 54 | 79**^**^** | 52 | 81**^**^** | 67 | 66 | 64 | 69 | 75 | 58 |
| III-IV (n=274) | 147 | 127 | 150 | 124 | 152 | 122 | 137 | 137 | 140 | 134 | 129 | 145 |
| Unknow (n=2) | 2 | 0 | 1 | 1 | 1 | 1 | 1 | 1 | 1 | 1 | 1 | 1 |
| **Histologic grade** |  |  |  |  |  |  |  |  |  |  |  |  |
| Low (n=21) | 11 | 10 | 4 | 17**^***^** | 5 | 16**^*^** | 9 | 12 | 17 | 4**^**^** | 15 | 6 |
| High (n=385) | 192 | 193 | 199 | 186 | 198 | 187 | 194 | 191 | 186 | 199 | 189 | 196 |
| Unknow (n=3) | 2 | 1 | 2 | 1 | 2 | 1 | 2 | 1 | 2 | 1 | 1 | 2 |
| **Depth of invasion** |  |  |  |  |  |  |  |  |  |  |  |  |
| pT1-T2 (n=124) | 55 | 69 | 51 | 73**^**^** | 57 | 67 | 62 | 62 | 61 | 63 | 66 | 58 |
| pT3-T4 (n=252) | 137 | 115 | 140 | 112 | 138 | 114 | 124 | 128 | 128 | 124 | 120 | 132 |
| Unknow (n=33) | 13 | 20 | 14 | 19 | 10 | 23 | 19 | 14 | 16 | 17 | 19 | 14 |
| **Lymphatic met.** |  |  |  |  |  |  |  |  |  |  |  |  |
| No (n=237) | 116 | 121 | 113 | 124**^*^** | 119 | 118 | 112 | 125 | 115 | 122 | 126 | 111 |
| Yes (n=130) | 73 | 57 | 77 | 53 | 73 | 57 | 73 | 57 | 69 | 61 | 57 | 73 |
| Unknow (n=42) | 16 | 26 | 15 | 27 | 13 | 29 | 20 | 22 | 21 | 21 | 22 | 20 |
| **Metastasis** |  |  |  |  |  |  |  |  |  |  |  |  |
| No (n=195) | 99 | 96 | 70 | 125 | 91 | 104 | 104 | 91 | 100 | 95 | 99 | 96 |
| Yes (n=11) | 4 | 7 | 4 | 7 | 4 | 7 | 7 | 4 | 5 | 6 | 5 | 6 |
| Unknow (n=203) | 102 | 101 | 131 | 72 | 110 | 93 | 94 | 109 | 100 | 103 | 101 | 102 |

Contingence table as follows.

| **Variables** | **miR-4444**  **Exp.** | | **miR-4652-5p**  **Exp.** | | **miR-4732-3p**  **Exp.** | | **miR-520f-5p Exp.** | | **miR-6734-5p Exp.** | | **miR-769-3p**  **Exp.** | |
| --- | --- | --- | --- | --- | --- | --- | --- | --- | --- | --- | --- | --- |
|  | Low  (n=205) | High  (n=204) | Low  (n=205) | High  (n=204) | Low  (n=205) | High  (n=204) | Low  (n=205) | High  (n=204) | Low  (n=205) | High  (n=204) | Low  (n=205) | High  (n=204) |
| **Gender** |  |  |  |  |  |  |  |  |  |  |  |  |
| Male (n=302) | 151 | 151 | 157 | 145 | 149 | 153 | 153 | 149 | 148 | 154 | 146 | 156 |
| Female (n=107) | 54 | 53 | 48 | 59 | 56 | 51 | 52 | 55 | 57 | 50 | 59 | 48 |
| **Age** |  |  |  |  |  |  |  |  |  |  |  |  |
| ≤65 (n=162) | 76 | 86 | 84 | 78 | 80 | 82 | 76 | 86 | 80 | 82 | 86 | 76 |
| >65 (n=247) | 129 | 118 | 121 | 126 | 125 | 122 | 129 | 118 | 125 | 122 | 119 | 128 |
| **Stage** |  |  |  |  |  |  |  |  |  |  |  |  |
| I-II (n=133) | 63 | 70 | 64 | 69 | 65 | 68 | 61 | 72 | 60 | 73 | 61 | 72 |
| III-IV (n=274) | 140 | 134 | 140 | 134 | 139 | 135 | 143 | 131 | 145 | 129 | 143 | 131 |
| Unknow (n=2) | 2 | 0 | 1 | 1 | 1 | 1 | 1 | 1 | 0 | 2 | 1 | 1 |
| **Histologic grade** |  |  |  |  |  |  |  |  |  |  |  |  |
| Low (n=21) | 7 | 14 | 12 | 9 | 8 | 13 | 6 | 15 | 6 | 15 | 12 | 9 |
| High (n=385) | 196 | 189 | 191 | 194 | 195 | 190 | 199 | 186 | 199 | 186 | 192 | 193 |
| Unknow (n=3) | 2 | 1 | 2 | 1 | 2 | 1 | 0 | 3 | 0 | 3 | 1 | 2 |
| **Depth of invasion** |  |  |  |  |  |  |  |  |  |  |  |  |
| pT1-T2 (n=124) | 55 | 69 | 61 | 63 | 62 | 62 | 54 | 70 | 58 | 66 | 56 | 68 |
| pT3-T4 (n=252) | 132 | 120 | 129 | 123 | 130 | 122 | 136 | 116 | 131 | 121 | 136 | 116 |
| Unknow (n=33) | 18 | 15 | 15 | 18 | 13 | 20 | 15 | 18 | 16 | 17 | 13 | 20 |
| **Lymphatic met.** |  |  |  |  |  |  |  |  |  |  |  |  |
| No (n=237) | 113 | 124 | 107 | 130**^**^** | 118 | 119 | 123 | 114 | 120 | 117 | 124 | 113 |
| Yes (n=130) | 70 | 60 | 79 | 51 | 67 | 63 | 66 | 64 | 60 | 70 | 68 | 62 |
| Unknow (n=42) | 20 | 20 | 19 | 23 | 20 | 22 | 16 | 26 | 25 | 17 | 13 | 29 |
| **Metastasis** |  |  |  |  |  |  |  |  |  |  |  |  |
| No (n=195) | 94 | 101 | 88 | 107 | 91 | 104 | 99 | 96 | 89 | 102 | 93 | 102 |
| Yes (n=11) | 6 | 5 | 6 | 5 | 5 | 6 | 4 | 7 | 4 | 7 | 3 | 8 |
| Unknow (n=203) | 105 | 98 | 111 | 92 | 109 | 94 | 102 | 101 | 112 | 91 | 109 | 94 |

Abbreviations: Exp., Expression level; Lymphatic met., Lymphatic metastasis.

*P<0.05; **P<0.01; ***P<0.001; Fisher's exact test.
