# Supplementary figures and images for: Identification of a dysregulated CircRNA-associated gene signature for predicting prognosis, immune landscape, and drug candidates in bladder cancer
Source: Front Oncol. 2022 Oct 10;12:1018285. doi: 10.3389/fonc.2022.1018285 (PMC9589509; doi:10.3389/fonc.2022.1018285)

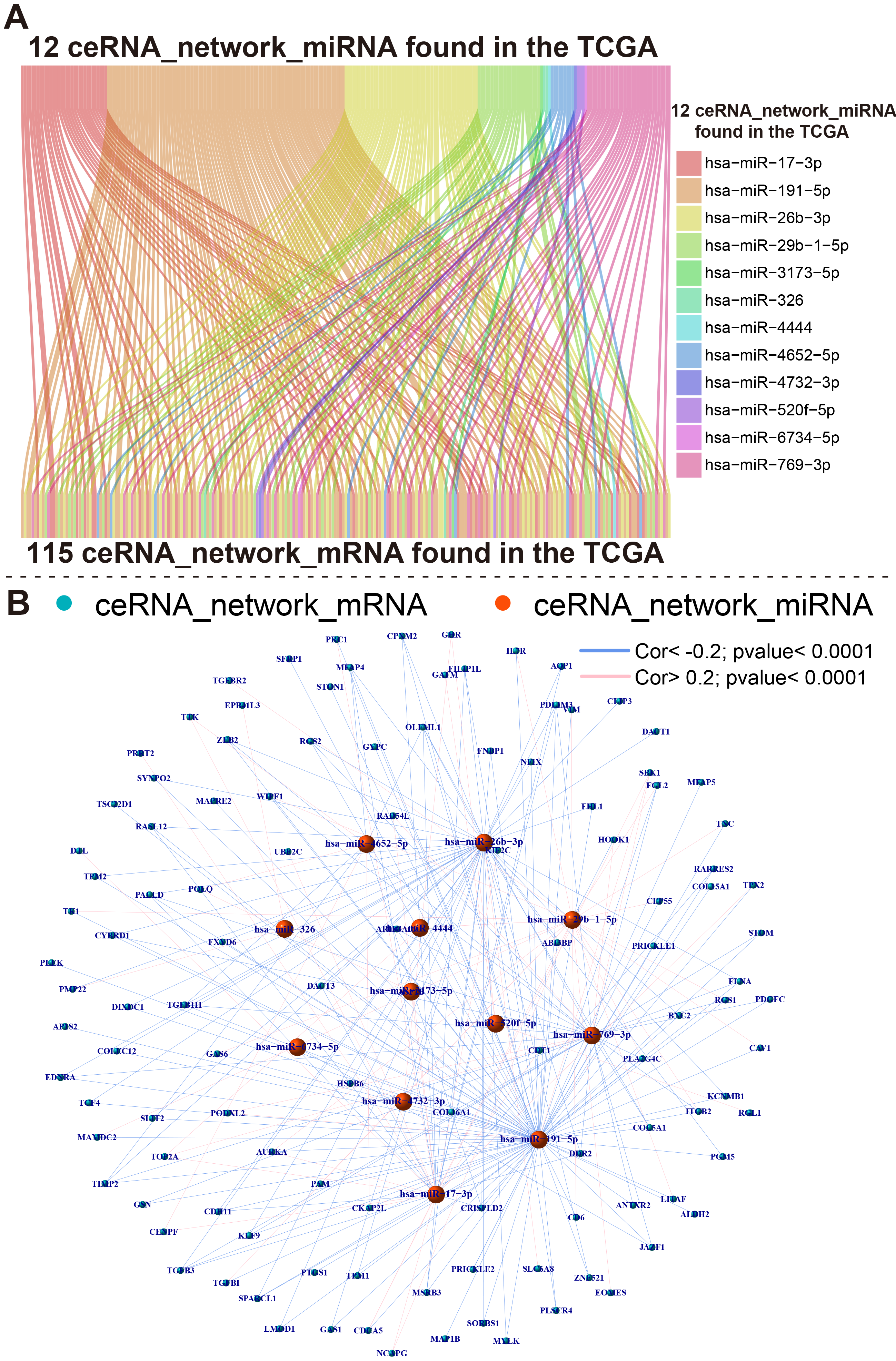

Supplement: Supplementary Figure 1 — A correlation between 12 DE-miRNAs and 115 DE-mRNAs form TCGA dataset. (A) The Sankey diagram was plotted according to the correspondence between 12 DE-miRNAs and 115 DE-mRNAs. (B) A correlation analysis of 12 DE-miRNAs and 115 DE-mRNAs expression data. Red lines indicate positive co-expression relationships while blue lines indicate negative co-expression relationships. miRNA, microRNA; differentially expressed-, DE-. [file Image_1.tif]

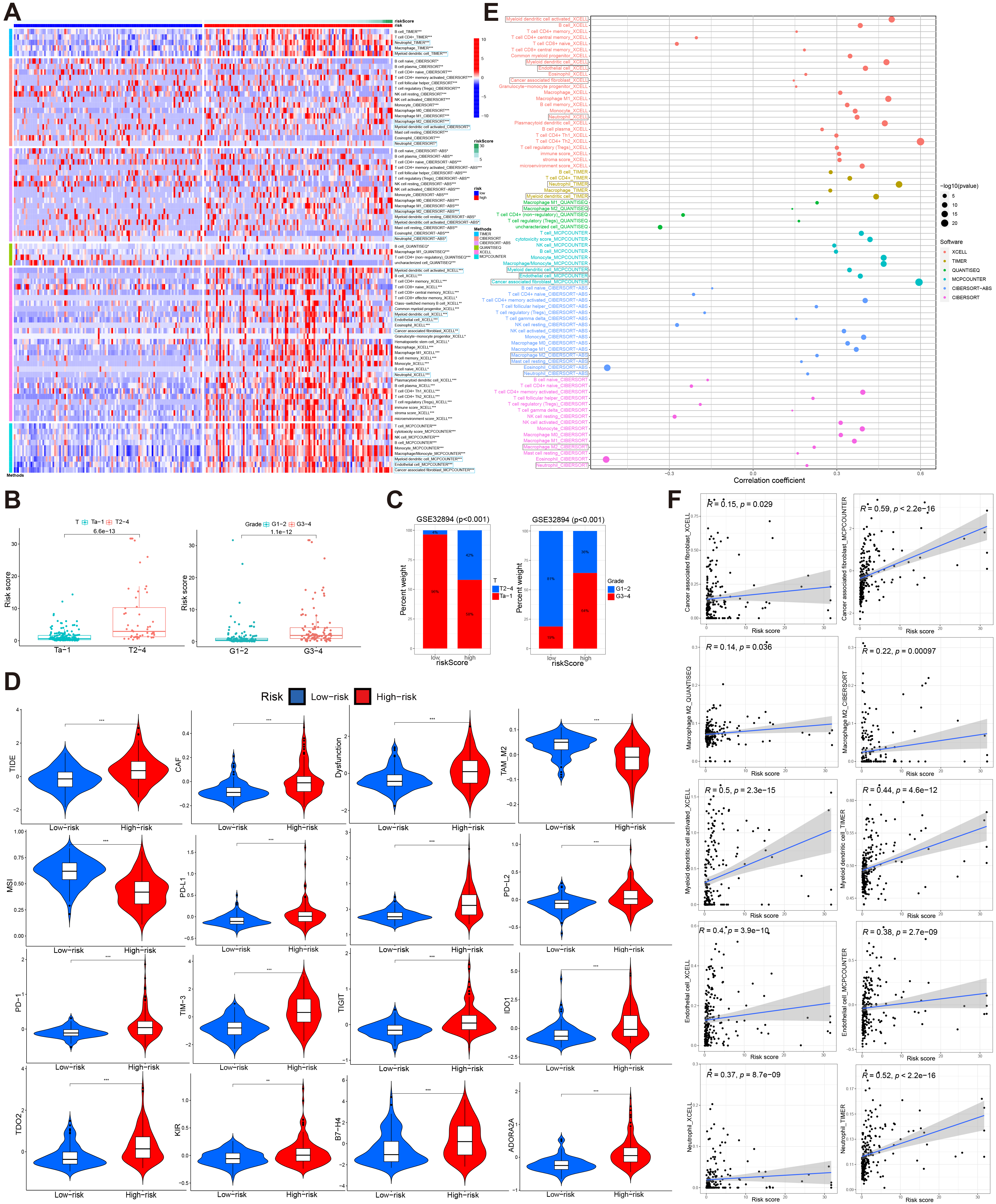

Supplement: Supplementary Figure 2 — We carried out an immune cell infiltration analysis and the efficacy prediction of immunotherapy based on ceRNA-associated gene signature in the test GSE32894 datasets. (A) Heat-mapping demonstrated many significant differences in immune cell infiltration between the high- and low-risk groups. (B, C) Wilcox and chi-square tests were used to assess the association between the ceRNA-associated gene risk signature and clinicopathological features, including the depth of tumor invasion and tumor grade. (D) Based on riskscore, the TIDE algorithm and the expression of immune checkpoint genes were then employed to potentially assess responses to immune checkpoint inhibitor. (E, F) The Spearman correlation between tumor-infiltrating immune cells and riskscore. [file Image_2.tif]

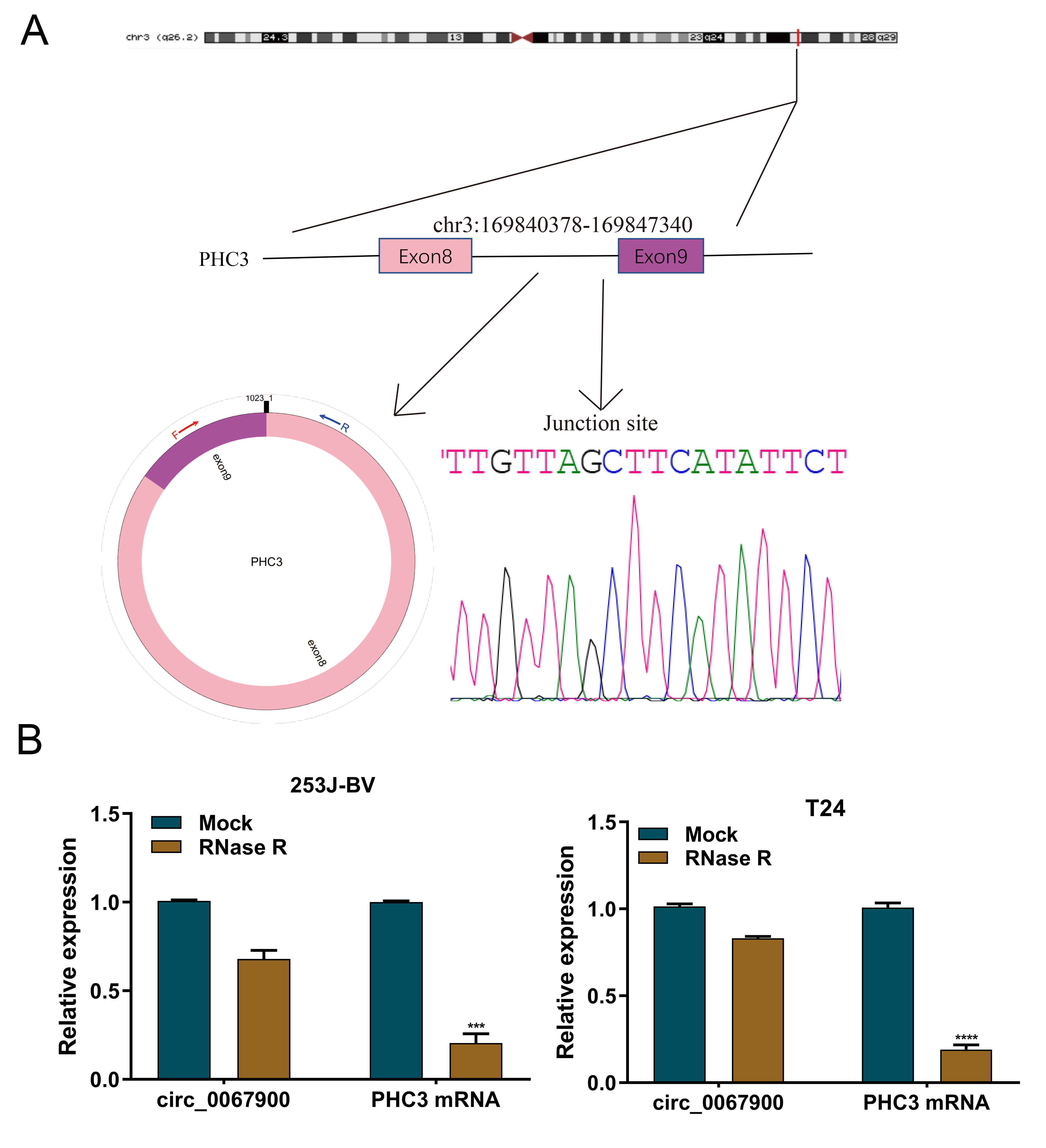

Supplement: Supplementary file 6 [file Image_3.tif]
